# Phospho-Akt overexpression is prognostic and can be used to tailor the synergistic interaction of Akt inhibitors with gemcitabine in pancreatic cancer

**DOI:** 10.1186/s13045-016-0371-1

**Published:** 2017-01-06

**Authors:** Daniela Massihnia, Amir Avan, Niccola Funel, Mina Maftouh, Anne van Krieken, Carlotta Granchi, Rajiv Raktoe, Ugo Boggi, Babette Aicher, Filippo Minutolo, Antonio Russo, Leticia G. Leon, Godefridus J. Peters, Elisa Giovannetti

**Affiliations:** 1 Department of Medical Oncology VU University Medical Center, Cancer Center Amsterdam, CCA room 1.52, De Boelelaan 1117, 1081 HV Amsterdam, The Netherlands; 2 Department of Surgical, Oncological and Oral Sciences, Section of Medical Oncology, University of Palermo, Palermo, Italy; 3 Metabolic syndrome Research center, School of Medicine, Mashhad University of Medical Sciences, Mashhad, Iran; 4 Cancer Pharmacology Lab, AIRC Start Up Unit, University of Pisa, Pisa, Italy; 5 Department of Pharmacy, University of Pisa, Pisa, Italy; 6 Department of Surgery, University of Pisa, Pisa, Italy; 7 Æterna Zentaris GmbH, Frankfurt am Main, Frankfurt, Germany

**Keywords:** Pancreatic ductal adenocarcinoma, Akt, Synergism, Gemcitabine

## Abstract

**Background:**

There is increasing evidence of a constitutive activation of Akt in pancreatic ductal adenocarcinoma (PDAC), associated with poor prognosis and chemoresistance. Therefore, we evaluated the expression of phospho-Akt in PDAC tissues and cells, and investigated molecular mechanisms influencing the therapeutic potential of Akt inhibition in combination with gemcitabine.

**Methods:**

Phospho-Akt expression was evaluated by immunohistochemistry in tissue microarrays (TMAs) with specimens tissue from radically-resected patients (*n* = 100). Data were analyzed by Fisher and log-rank test. In vitro studies were performed in 14 PDAC cells, including seven primary cultures, characterized for their *Akt1* mRNA and phospho-Akt/Akt levels by quantitative-RT-PCR and immunocytochemistry. Growth inhibitory effects of Akt inhibitors and gemcitabine were evaluated by SRB assay, whereas modulation of Akt and phospho-Akt was investigated by Western blotting and ELISA. Cell cycle perturbation, apoptosis-induction, and anti-migratory behaviors were studied by flow cytometry, AnnexinV, membrane potential, and migration assay, while pharmacological interaction with gemcitabine was determined with combination index (CI) method.

**Results:**

Immunohistochemistry of TMAs revealed a correlation between phospho-Akt expression and worse outcome, particularly in patients with the highest phospho-Akt levels, who had significantly shorter overall and progression-free-survival. Similar expression levels were detected in LPC028 primary cells, while LPC006 were characterized by low phospho-Akt. Remarkably, Akt inhibitors reduced cancer cell growth in monolayers and spheroids and synergistically enhanced the antiproliferative activity of gemcitabine in LPC028, while this combination was antagonistic in LPC006 cells. The synergistic effect was paralleled by a reduced expression of ribonucleotide reductase, potentially facilitating gemcitabine cytotoxicity. Inhibition of Akt decreased cell migration and invasion, which was additionally reduced by the combination with gemcitabine. This combination significantly increased apoptosis, associated with induction of caspase-3/6/8/9, PARP and BAD, and inhibition of Bcl-2 and NF-kB in LPC028, but not in LPC006 cells. However, targeting the key glucose transporter Glut1 resulted in similar apoptosis induction in LPC006 cells.

**Conclusions:**

These data support the analysis of phospho-Akt expression as both a prognostic and a predictive biomarker, for the rational development of new combination therapies targeting the Akt pathway in PDAC. Finally, inhibition of Glut1 might overcome resistance to these therapies and warrants further studies.

**Electronic supplementary material:**

The online version of this article (doi:10.1186/s13045-016-0371-1) contains supplementary material, which is available to authorized users.

## Background

Pancreatic ductal adenocarcinoma (PDAC) is among the most lethal solid tumors. Despite extensive preclinical and clinical research, the prognosis of this disease has not significantly improved, with a 5-year survival rate around 7% [1]. This dismal outcome can partially be explained by the lack of biomarkers for screening and diagnosis at earlier stages, and by the resistance to most currently available chemotherapy regimens. This resistance has been attributed to both the desmoplastic tumor microenvironment and to the strong inter- and intra-tumor heterogeneity in terms of complexity of genetic aberrations and the resulting signaling pathway activities, as well as to resistance mechanisms that quickly adapt the tumor to drugs [2].

Oncogenic *KRAS* signaling is the main driving force behind PDAC. Activating KRAS mutations occur early, followed by loss of *p16*, and then later, inactivation of *TP53* and *SMAD4* [3, 4]; however, targeting these events has proven to be very difficult. Conversely, the phosphatidylinositol-3 kinase (PI3K)/Akt downstream pathway represents an exciting new target for therapeutic intervention, especially because it emerged among the core signaling pathways in PDAC [5, 6], and several known inhibitors are currently in clinical trials (www.clinicaltrials.gov).

In particular, the serine/threonine kinase Akt, which is coded in three highly homologous isoforms (Akt1, Akt2, and Akt3), is overexpressed in more than 40% of PDAC patients [7]. Mechanisms underlying aberrant Akt activation in cancer include direct alterations such as mutations, amplification, or overexpression, but also activation of upstream signaling events, such as activation of HER-2/neu signaling or PTEN mutation/loss [8–11].

The PI3K/Akt pathway plays a key role in cell proliferation, survival, and motility [12]. Deregulation of components involved in this pathway could confer resistance to chemotherapy [13, 14], while blockage of Akt signaling results in programmed cell death and inhibition of tumor growth [15, 16]. Activation of Akt is a frequent event in PDAC and has been correlated to its poor prognosis [17, 18].

Several inhibitors of Akt are under investigation, but three are the farthest along and showed the most promise in early clinical research: the pan-Akt and PI3K inhibitor perifosine (KRX-0401, Aeterna Zentaris/Keryx), the allosteric pan-Akt inhibitor MK-2206 (Merck), and the dual PI3K/mTOR inhibitor dactolisib (NVP-BEZ235, Novartis).

In particular, the synthetic oral alkylphospholipid perifosine [19, 20] has been evaluated in clinical trials for several tumors, including colon [21], breast [22], head and neck, and prostate cancer [23, 24]. Unfortunately, it failed the phase III clinical trials for treatment of colon cancer and relapsed refractory multiple myeloma (www.clinicaltrials.gov). These failures, together with the disappointing response rates to perifosine as a single agent in most solid tumors, including PDAC, prompt further studies into its mechanism of action [6] as well as on synergistic combinations.

Perifosine prevents translocation of Akt to the cell membrane by blocking the pleckstrin homology (PH) domain of Akt [25] leading to inactivation of downstream pathway and inhibition of cell proliferation. Previous studies demonstrated perifosine activity against different cancer types, in vitro and in vivo [26]. Recently, Pinton and collaborators showed that perifosine inhibited cell growth of malignant pleural mesothelioma cells by affecting EGFR and c-Met phosphorylation [27]. Another study showed that perifosine decreased the *AEG-1* gene expression along with inhibition of Akt/GSK3/c-Myc signaling pathway in gastric cancer [28]. Perifosine and curcumin synergistically increased the intracellular level of reactive oxygen species and ceramide, and downregulated the expression of cyclin-D1 and Bcl-2 in colorectal cancer cells [29]. Finally, perifosine also inhibits the anti-apoptotic mitogen-activated protein kinase (MAPK) pathway and modulates the balance between the MAPK and pro-apoptotic stress-activated protein kinase (SAPK/JNK) pathways, thereby inducing apoptosis [30].

The aims of current study were to investigate the expression of phospho-Akt in PDAC tissues and cells, and to evaluate the effects of growth inhibition by Akt inhibitors, using PDAC cell lines and primary cultures growing as monolayer or as spheroids. Moreover, we characterized several key factors, affecting cell cycle perturbation, apoptosis induction, as well as inhibition of cell migration and invasion and modulation of key factors in glucose metabolism in PDAC cells exposed to perifosine and perifosine/gemcitabine combination.

## Methods

### Tissue microarrays (TMAs), immunohistochemistry (IHC), and immunocytochemistry (ICC)

Phospho-Akt protein expression was evaluated in slides from four formalin-fixed, paraffin-embedded PDAC-specific TMAs build with neoplastic cores from a cohort of radically resected patients (*n* = 100), using the TMA Grand Master (3DHistec, Budapest, Hungary) instrument, and stained according to standard procedures with the EP2109Y rabbit monoclonal antibody (1:50 dilution; Abcam, Cambridge, UK). Visualization was obtained with BenchMark Special Stain Automation system (Ventana Medical Systems, Tucson, AZ). Two pathologists reviewed all the slides, assessing the amount of tumor and tissue loss, background staining, and overall interpretability before the phospho-Akt reactivity evaluation. Staining results were evaluated using a computerized high-resolution acquisition system (D-Sight, Menarini, Florence, Italy), including the analysis of positive cells number and staining intensity which resulted in values expressed as arbitrary units (a.u.). All patients have provided a written informed consent. This study was approved by the Local Ethics Committee of the University of Pisa. Date of approval: July 3, 2013 (file number 3909).

For ICC, the cells were grown in a Chamber Slides System (Lab-Tek, Collinsville, IL). After 24 h, the cells were fixed with 70% ethanol for 10 min, followed by incubation with the antibody described above (4 °C overnight, 1:30 dilution in PBS). Cells were stained with the avidin-biotin-peroxidase complex (UltraMarque HRP Detection, Greenwood, AR). Negative controls were obtained by replacing the primary antibody with PBS. The sections were reviewed and scored using a digital system based on staining intensity and on the number of positively stained cells, as described above.

### Drugs and chemicals

Perifosine was provided by Æterna Zentaris Inc. (Frankfurt am Main, Germany), NVP-BEZ235 was purchased from Selleck Chemicals (Houston, TX), while gemcitabine and MK-2206 were generous gifts from Eli-Lilly (Indianapolis, IN) and Merck (Whitehouse Station, NJ), respectively. The drugs were dissolved in Dimethyl sulfoxide (DMSO) or sterile water and diluted in culture medium before use. RPMI-1640 medium, foetal bovine serum (FBS), penicillin (50 IU/ml), and streptomycin (50 μg/ml) were from Gibco (Gaithersburg, MD). All other chemicals were purchased from Sigma-Aldrich (Zwijndrecht, The Netherlands).

### Cell cultures

Eight PDAC cell lines (PL45, MIA-PaCa2, HPAF-II, CFPAC-1, Bxpc3, HPAC, and PANC-1) and the human immortalized pancreatic duct epithelial-like cell line hTERT-HPNE were obtained from the American Type Culture Collection, whereas seven primary PDAC cultures (LPC006, LPC028, LPC033, LPC067, LPC111, LPC167, and PP437) were isolated from patients at the University Hospital of Pisa (Pisa, Italy), as described previously [31]. The cell lines were tested for their authenticity by PCR profiling using short tandem repeats by BaseClear (Leiden, The Netherlands). The cells were cultured in RPMI-1640, supplemented with 10% heat-inactivated FBS and 1% streptomycin/penicillin at 37 °C, and harvested with trypsin- EDTA in their exponentially growing phase.

### Quantitative reverse-transcriptase polymerase-chain-reaction (qRT-PCR)

Total RNAs were extracted from cells using the TRI REAGENT-LS (Invitrogen, Carlsbad, CA), according to the manufacturer’s protocol. RNA was also extracted from seven primary tumors, after laser micro-dissection with a Leica-LMD7000 instrument (Leica, Wetzlar, Germany), using the QIAamp RNA Micro Kit (Qiagen, Hilden, Germany), as described [31].

RNA yield and purity were checked at 260 to 280 nm with NanoDrop-1000 Detector (NanoDrop Technologies, Wilmington, DE). One microgram of RNA was reverse-transcribed using the DyNAmo Synthesis Kit (Thermo Scientific, Vantaa, Finland). qRT-PCR was performed with specific TaqMan® primers and probes for *Akt1*, *human equilibrative nucleoside transporter-1* (*hENT1*), *deoxycytidine kinase (dCK)*, *cytidine deaminase* (*CDA*), *ribonucleotide reductase subunit-M1* (*RRM1*), *and subunit-M2* (*RRM2*), *E-cadherin*, and the *glucose transporter 1* (*SLC2A1*/*Glut1*) which were obtained from Applied Biosystems TaqMan Gene expression products (Hs00920503_m1, Hs01085706_m1, Hs00984403_m1, Hs01040726_m1, Hs00156401_m1, Hs00168784_m1, Hs01072069_g1, Hs01023894_m1, and Hs00892681_m1). The cDNA was amplified using the ABI-PRISM 7500 instrument (Applied Biosystems, Foster City, CA). Gene expression values were normalized to β-actin, using a standard curve of cDNAs obtained from Quantitative PCR Human Reference RNA (Stratagene, La Jolla, CA), as described earlier [32].

### Growth inhibition studies

The cell growth inhibitory effects of perifosine, MK-2206 and NVP-BEZ235 were evaluated in the PANC-1, LPC028, and LPC006 cells. Further studies evaluated perifosine and gemcitabine combination in CFPAC-1, PANC-1, LPC028, and LPC006 cells. These cells were treated for 72 h with perifosine (1–500 μM), gemcitabine (1–500 nM), and simultaneous combination at a fixed ratio based on IC50 (i.e., concentration of a drug required for 50% inhibition of cell growth) of each drug. The plates were then processed for the sulforhodamine-B assay, as described [32].

### Evaluation of synergistic/antagonistic interaction with gemcitabine

The pharmacological interaction between perifosine and gemcitabine was evaluated by the median drug effect analysis method as described previously [32]. In this regard, the combination index (CI) was calculated to compare cell growth inhibition of the combination and each drug alone. Data analysis was carried out using CalcuSyn software (Biosoft, Oxford, UK).

### Effects on multicellular spheroids

LPC006 and LPC028 spheroids were established by seeding 10^4^ cells per ml in DMEM/F12 + GlutaMAX-I (1:1) with insulin-transferrin-selenium (1:1000, Invitrogen), in 24-well ultra-low attachment plates (Corning Incorporated, NY). The cytotoxic effects were evaluated by measuring the size and number of spheroids with the inverted phase contrast microscope Leica-DMI300B (Leica, Wetzlar, Germany), taking 9 pictures for each well. Spheroid volume (V) was calculated from the geometric mean of the perpendicular diameters *D* = (D_max_ + D_min_)/2, as follows: *V* = (4/3) × π (D/2)3.

### Western blot

In order to evaluate the modulation of Akt1, phospho-Akt1, PARP, BAD, Bcl-2, NF-kB, and Glut1 protein expression in PDAC cells treated for 24 h with perifosine, gemcitabine, and their combination, Western blot analyses were executed as described previously using the Akt1 sc-5298 mouse monoclonal (Santa Cruz, Biotechnology, Santa Cruz, CA) and the EP2109Y rabbit monoclonal antibody (1:500 dilution; Abcam), PARP sc-8007 mouse monoclonal (1:500 dilution; Santa Cruz), BAD sc-8044 mouse monoclonal (1:500 dilution;Santa Cruz), Bcl-2 sc-7382 mouse monoclonal (1:500 dilution; Santa Cruz), NF-kB sc-114 rabbit polyclonal (1:500 dilution; Santa Cruz), and Glut1 sc-1605 goat polyclonal (1:500 dilution; Santa Cruz) [33]. Briefly, 40 μg of proteins was separated on a 10% SDS-polyacrylamide gel and transferred onto polyvinylidene difluoride (PVDF) membrane (Immobilion®-FL, Millipore, Billerica, MA). The membrane was incubated overnight with mouse and rabbit anti-Akt1, anti-phospho-Akt1, as described above, as well as with mouse anti-BAD, anti-Bcl-2, anti-PARP, with rabbit anti-NF-kB (1:1000, diluted in the blocking solution; all from Santa Cruz Biotechnology, Santa Cruz, CA), goat anti-Glut-1 (ab652, 1:500, diluted in the blocking solution, from Abcam, Cambridge, UK), and mouse anti-β-actin (1:10000; Sigma–Aldrich). The secondary antibodies were goat anti-rabbit-InfraRedDye® 800 Green and goat anti-mouse InfraRedDye® 680 Red (1:10000, Westburg, Leusden, The Netherlands). Fluorescent proteins were monitored by an Odyssey Infrared Imager (LI-COR Biosciences, Lincoln, NE), equipped with Odyssey 2.1 software to perform a semi-quantitative analysis of the bands.

### Akt and phospho-Akt analysis by enzyme linked immunosorbent (ELISA) assay

To investigate the inhibitory effects of perifosine on Akt [pS473] and [Thr308] phosphorylation, specific ELISA assays were performed using the Pierce AKT Colorimetric In-cell ELISA Kit (Thermo Scientific, Rockford, IL), which has a sensitivity approximately twofolds greater than Western blotting. The levels of Akt and phospho-Akt were measured in cells seeded in a 96-well-plate at a density of 10^5^ cells per well, and treated for 4 or 24 h with perifosine, gemcitabine, and their combination at IC_50_ values. The absorbance was measured in a Synergy HT Multi-Detection Microplate Reader (BioTek, Bad Friedrichshall, Germany) at a wavelength of 450 nm.

### In vitro migration and invasion assays

The ability of perifosine and its combination with gemcitabine and MK-2206 and its combination with gemcitabine to inhibit the migratory behaviour of PDAC cells was investigated by in vitro migration assay, as described [31]. The cells were exposed to the drugs at their IC_50s_. Images were taken at the beginning of the exposure (time 0), with those taken after 4, 6, 8, 20, and 24 h. Transwell chambers with polycarbonate membranes, and 8 μm pores were used for invasion assays. These assays were carried out through coated transwell filters, with 100 μl of 0.1 mg/mL collagen I solution. A total of 10^5^ cells were plated on the upper side of the filter and incubated with the drugs at IC_50_ concentrations in RPMI-1640 medium. After 24 h, cells migrated into the lower side were fixed with paraformaldehyde and stained with Giemsa in 20% methanol. The filters were photographed and cells were counted.

### Analysis of cell-cycle and cell death

To investigate the effect of drugs on modulation of cell cycle, LPC028, LPC006, CFPAC-1, and PANC-1 cells were treated for 24 h with gemcitabine, perifosine, and their combination at IC_50_ concentrations. Cells were stained by propidium iodide (PI) and cell cycle modulation was evaluated using a FACSCalibur flow cytometer (Becton Dickinson, San José, CA), equipped with the CELLQuest software for data analysis.

The ability of gemcitabine, perifosine, and its combination with gemcitabine to induce cell death was evaluated by measuring sub-G1 regions during cell cycle analysis, as described above. Apoptosis induction was also assessed by 3,3′-dihexyloxacarbocyanine iodide (DiOC) labelling. DiOC is a lipophilic and green fluorescent dye, which can pass the plasma membrane, without being metabolized by the cell, and accumulate at the membrane of mitochondria of living cells. Shortly, the cells were stained with DiOC for 30 min, and analysed by FACSCalibur, as described [34]. Additional studies were performed with the Annexin-V/PI assay, plating the cells in 6-well-plates at a density of 1.5 × 10^5^. After 24 h, the cells were treated with the drugs at their IC_50_, followed by 24-h incubation. Then, the cell pellets were re-suspended in 100 mL of ice-cold binding buffer (0.1 M Hepes/NaOH (pH = 7.4), 1.4 M NaCl, 25 mm CaCl_2_). The staining was performed according to the manufacturer’s instructions (Annexin-V/PI detection Kit-I, Becton Dickinson). Cells were stained by 5 μL Annexin V-FITC and 5 μL PI. Samples were gently vortexed and incubated for 15 min at room temperature. Then, 400 μL of binding buffer was added to the cells. The samples were analyzed by FACSCalibur using excitation/emission wavelengths of 488/525 and 488/675 nm for Annexin-V and PI, respectively.

### Caspase activity assay

The effects of perifosine, gemcitabine and their combination on the activity of caspase-3, -6, -7, -8, -9 were determined by specific fluorometric assay kits (Zebra Bioscience, Enschede, The Netherlands), according to the manufacturer’s instructions. Briefly, 10^6^ LPC006, LPC028, CFPAC-1, and PANC-1 cells were exposed to the drugs for 24 h at their IC_50s_. Fluorescence was measured at 350 nm excitation and 460 nm emission (Spectrafluor Tecan, Salzburg, Austria). Relative caspase activity was normalized with respect to the untreated cells.

### Analysis of modulation of Glut1 by flow cytometry

To quantitatively detect the expression of membrane-bound Glut1, cells were fixed with 80% ethanol, incubated with anti-Glut1 antibody (Abcam), and then stained with the appropriate FITC-conjugated anti-rabbit IgG antibody (BD Pharmingen™, BD Biosciences, San Jose, CA). Quantification of FITC fluorescence intensity was performed using a FACSCanto flow cytometer (BD Biosciences).

### Evaluation of the cytotoxic and pro-apoptotic effects inhibition of Glut1 inhibition combined with Akt inhibitors

The Akt signaling is involved in the modulation of Glut1 expression/localization, and a recent study showed that increased glucose metabolism was associated to resistance to the tyrosine kinase inhibitor axitinib, and this resistance was overcame by Glut1 silencing [35]. Therefore, we performed additional cytotoxicity studies using the novel Glut1 inhibitor PGL13. This compound was tested in the LPC006 cells, at a concentration of 30 μM, which effectively reduced glucose influx in previous studies [36, 37]. The cells were exposed to PGL13 for 72 h, alone or in combination with IC_50_ concentration values of perifosine, gemcitabine, and their combination. Cell growth inhibition was then assessed by counting the cells after staining with trypan blue, in comparison to untreated cells. Parallel evaluation of apoptosis induction was performed by fluorescence microscopy with bisbenzimide staining, as described previously [33].

### Statistical analysis

All experiments were performed in triplicate and repeated at least twice. Data were expressed as mean values ± SEM and analyzed by Student’s *t* test or ANOVA followed by Tukey’s multiple comparison test. For the analysis of the correlation of phospho-Akt expression and clinical data, the overall survival (OS), and progression-free-survival (PFS) were calculated from the date of pathological diagnosis (i.e., the date of surgery) to the date of death and tumor progression, respectively. OS and PFS curves were constructed using Kaplan-Meier method, and differences were analyzed using log-rank test. Data were analyzed using SPSS v.20 statistical software (IBM, Chicago). Statistical significance was set at *P* < 0.05.

## Results

### Correlation with outcome and phospho-Akt and Akt1 mRNA expression in PDAC tissues and cells

**Fig. 1:**
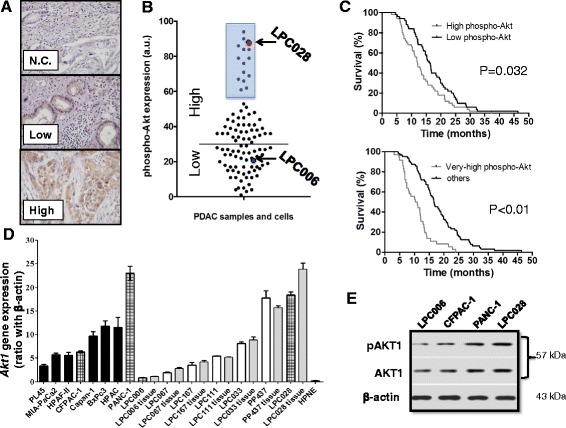
Akt/phospho-Akt expression in PDAC tissues and cells. **a** Representative examples (original magnification, ×40) showing the variable expression of phospho-Akt in paraffin-embedded PDAC samples collected in four TMAs (with 4 cores for each of the 100 patients). *N.C*., negative control. **b** Expression values of phospho-Akt observed across the cohort of PDAC patients, obtained by digital quantification. Phospho-Akt showed positive cytoplasmic and nuclear staining in most tissue sections, with intense staining in 14 out of 100 samples. The staining intensities of the LPC028 and LPC006 cells were included in the very high and low Akt expression groups, respectively. **c** Kaplan–Meier survival curves according to the expression of phospho-Akt in 100 radically resected PDACs, showing that patients with high expression (*upper panel*) and very high expression (*lower panel*) of phospho-Akt had a significantly shorter survival compared to patients with low phospho-Akt expression. **d** *Akt1* mRNA expression in ATCC cell lines (*black bars*), primary tumor cultures (*white bars*), and their originator tissues (*gray bars*). *Dashed bars* identify the cells that were selected for further in vitro studies; **e** Representative Western blot pictures of phospho-Akt1 and Akt1 expression in LPC006, CFPAC-1, PANC-1, and LPC028 cells. *Columns*, mean values obtained from three independent experiments, *bars*, SEM

The protein expression of phospho-Akt was successfully evaluated by IHC in 100 human PDACs collected in two TMAs. The main clinical characteristics of these patients are reported in the Table 1. IHC showed a variable protein expression with some specimens characterized by a strong and diffuse staining, while other tissues had only a few scattered positive cells with a weak staining (as exemplified by the middle and lower panels in the Fig. 1a, respectively). Patients were categorized according to their high versus low phospho-Akt expression compared to the median value (30 a.u.) calculated by digital scoring (Fig. 1b, black line). No association was observed between phospho-Akt and age, sex, grading, resection, and lymph node infiltration (data not shown). Patients with low phospho-Akt expression had a median OS of 16.2 months (95% CI, 14.8–20.1), while patients with a high expression had a median OS of 12.0 months (95% CI, 9.0–14.9, *P* = 0.03, Fig. 1c, upper panel). However, only a trend toward a significant association was found between phospho-Akt expression and PFS (*P* = 0.08, Additional file 1: Figure S1a).

**Table 1:** Outcome according to clinical characteristics in the 100 PDAC patients enrolled in the present study

Characteristics		N (=%)	OS months (95% CI)	P
No. patients	All	100	14.0 (12.1–15.8)	
Age, years	≤65	43	15.2 (13.3–16.8)	0.361
	>65	57	14.1 (11.1–17.0)	
Sex	Male	47	13.0 (11.1–14.9)	0.814
	Female	53	15.0 (11.9–18.1)	
Resection status	R0	56	15.2 (12.3–21.6)	0.474
	R1	44	13.5 (11.2–31.0)	
Lymph node	No	10	18.5 (7.6–32.4)	0.521
	Yes	90	14.2 (12.2–15.9)	
Grading	1–2	36	15.5 (11.5–18.1)	0.097
	3	64	12.1 (8.4–15.8)

An additional analysis was performed categorizing the patients with respect to a threshold expression of 57 a.u., which identified 14 cases with higher expression compared to all the others (defined as *very high* phospho-Akt expression, Fig. 1b, blue square). Using these categories, we observed a significant correlation between high phospho-Akt protein expression and both significantly shorter OS (*P* < 0.01, Fig. 1c, lower panel), and PFS (Additional file 1: Figure S1b).

Parallel ICC studies revealed that the LPC006 cells had a significantly lower phospho-Akt expression compared to LPC028 cells, which were indeed included in the category of *low* and very high phospho-Akt expression, respectively (Fig. 1b, blue and red circles). The mRNA expression of *Akt1* was detectable in all PDAC cells by qRT-PCR, as well as in the originator tissues of the primary tumor cell cultures. This expression value differed among the cells, ranging from 0.9 arbitrary unit (a.u.) in LPC006 cells to 24.0 a.u. in LPC028 and PANC-1 cells (Fig. 1d). The mean and median expression in the tumor cells (8.7 ± 0.2 and 8.4 a.u., respectively) were significantly higher (*P* < 0.01) than the expression detected in hTERT-HPNE cells (0.3 a.u.). Notably, *Akt1* gene expression in the seven primary tumor cells and their laser-microdissected originator tumors showed a similar pattern and were highly correlated with Spearman analysis (*R* ^2^ > 0.9, *P* < 0.05), suggesting that these cells represent optimal preclinical models for our pharmacological studies. Moreover, Western blot analysis revealed that the LPC006 and CFPAC-1 cells had a lower phospho-Akt1/Akt1 ratio (0.3 and 0.6 a.u., respectively) expression compared to PANC-1 (0.8) and LPC028 (1.1) cells (Fig. 1e).

Therefore, we selected for further studies two primary cell cultures (LPC006 and LPC028) which were representative of low and very high expression values, as well as two cell lines, PANC-1 and CFPAC-1, with high and intermediate expression values of Akt1 mRNA, respectively.

### Perifosine inhibits cell growth and interacts synergistically with gemcitabine in PDAC cells with high expression of phospho-Akt

**Fig. 2:**
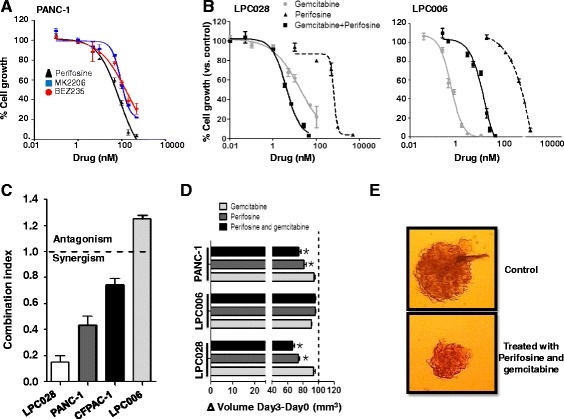
Inhibition of cell proliferation in PDAC cells. **a** Growth inhibitory effects in PANC-1 cells after 72 h exposure to perifosine, MK-2206 and NVP-BEZ235. **b** Growth inhibitory effects after 72 h exposure to perifosine, gemcitabine, or their combination at a fixed ratio based on IC_50_ values in LPC028 and LPC006 cells. On the *X* axis, the drug concentrations for the combination are referred to gemcitabine. **c** Mean CI of the perifosine/gemcitabine combination. CI values at FA of 0.5, 0.75. and 0.9 were averaged for each experiment, and this value was used to calculate the mean between experiments, as explained in the Methods section. **d** Effect of perifosine and gemcitabine. and their combination, at IC_50_ values, on the volumes of PDAC spheroids after 72 h exposure. **e** Representative images of untreated spheroid versus spheroid treated with perifosine and gemcitabine (original magnification, ×40). *Columns* and *points* mean values obtained from three independent experiments, *bars*, SEM; *Significantly different from controls

The cytotoxic activity of three different Akt inhibitors (perifosine, MK-2206, and NVP-BEZ235) was evaluated in the PANC-1 cell line (Fig. 2a). All these compounds caused a concentration-dependent inhibition of proliferation, with IC_50_ values ranging from 5.1 (perifosine) to 15.8 μM (NVP-BEZ235). Higher IC_50_ values were obtained in the LPC006 cells, i.e., 22.5, 31.7 and 45.5 μM for perifosine, NVP-BEZ235, and MK-2206 (Additional file 1: Figure S2), respectively. According to the lowest IC_50_ values detected in these assays, we selected perifosine for the following studies on the pharmacological interaction of Akt inhibitors with gemcitabine.

The cell growth inhibitory effects of perifosine, gemcitabine, and their combination in LPC028 and LPC006 cells are shown in Fig. 2b, while the data for CFPAC-1 and PANC-1 are reported in the Additional file 1: Figure S3. Since the CI method recommends a ratio of concentrations at which drugs are equipotent, combination studies were performed using fixed ratios with IC values at IC_50s_. Perifosine enhanced the antiproliferative activity of gemcitabine, especially in the LPC028 and PANC-1 cells, by decreasing the IC_50s_ of gemcitabine from 4.3 ± 1.1 and 17.2 ± 2.1 nM to 1.4 ± 0.5 and 4.0 ± 1.1 nM, respectively. The median drug-effect analysis revealed a slight-to-moderate synergism in CFPAC-1, and a strong synergism in the PANC-1 and LPC028 cells, with CI values of 0.8, 0.5, and 0.2, respectively (Fig. 2c). Conversely, the combination of perifosine and gemcitabine was antagonistic in the LPC006 cells (CI > 1.2). To evaluate whether these effects were observed also in three-dimensional (3D) models and investigate the mechanisms underlying these different interactions, several biochemical analyses were performed, as detailed below.

### Perifosine and its combination with gemcitabine reduce the size of PDAC spheroids

Previous studies illustrated that 3D culture models are generally more chemo-/radio-resistant than two-dimensional monolayer cell cultures, supporting their use for drug testing [38]. In order to explore whether perifosine would be active in 3D PDAC models, we evaluated this drug in spheroids of LPC006, LPC028, and PANC-1 cells.

Perifosine remarkably increased the disintegration of LPC028 and PANC-1 spheroids, which were significantly (*P* < 0.05) reduced in size compared to the untreated spheroids (Fig. 2d–e). The combination of perifosine with gemcitabine additionally reduced the size of the LPC028 and PANC-1 spheroids with respect to the spheroids treated with the single drugs. In contrast, no changes were observed in the LPC006 spheroids, further supporting the antagonistic interaction of perifosine with gemcitabine in this PDAC model.

### Modulation of phospho-Akt and gemcitabine determinants in PDAC cells

**Fig. 3:**
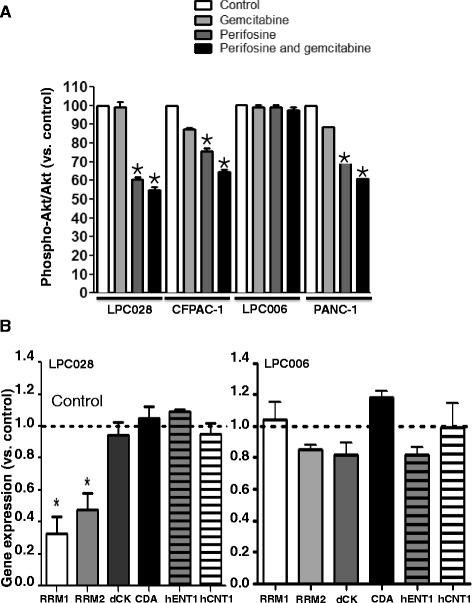
Modulation of phospho-Akt and gemcitabine determinants. **a** Effect of 24-h exposure to gemcitabine, perifosine or their combination, at IC_50_ values, on the expression of phospho-Akt, normalized to the expression of total Akt, as determined by ELISA. **b** Expression of gemcitabine key determinants in LPC028 (*left panel*) and LPC006 (*right panel*) cells treated with perifosine at IC_50_ versus untreated cells, as determined by qRT-PCR. *Columns* mean values obtained from three independent experiments, *bars*, SEM. *Dashed line*, values in untreated samples (Control). *Significantly different from controls

Perifosine inhibits the phosphorylation of Akt by blocking the PH-domain in different cancer cell lines [39], but no data have been reported yet on PDAC cells. Therefore, we evaluated the expression of phospho-Akt (at serine residue 473 (Ser473) and at threonine residues 308 (Thr308)), normalized to the total Akt levels, both in untreated cells and in cells treated with Akt inhibitors (perifosine and MK-2206), gemcitabine, and their combination. We observed a similar inhibition of the phosphorylation status after 4 or 24 h (Fig. 3a and Additional file 1: Figure S4) as well as in both residues (Additional file 1: Figure S5a, b). Perifosine significantly reduced the expression of p-Akt in LPC028, CFPAC-1, and PANC-1 cells (e.g., 40, 25, and 30% reduction, respectively). Regarding Ser473 phosphorylation, the combination of perifosine and gemcitabine was also able to significantly suppress Akt phosphorylation, with a degree of inhibition ranging from −35 (CFPAC-1 cells) to −45% (LPC028 cells). Conversely, both Ser473 and Thr308 phospho-Akt levels were not affected by perifosine, MK-2206, and their combination with gemcitabine in the LPC006 cells.

*RRM1* and *RRM2* encode for the catalytic and the regulatory subunits of ribonucleotide reductase and is a key molecular target of gemcitabine [40]. Previous studies demonstrated that the expression of *RRM2* is modulated by the Akt/c- MYC pathway [41]. However, the alterations in the expression or function of other enzymes, involved in the transport, metabolism, and catabolism of gemcitabine can also lead to resistance (e.g., decreased dCK or increased CDA expression [40]). Therefore, we evaluated the mRNA expression of several gemcitabine determinants in the LPC006, LPC028 and PANC-1 cells. As shown in Fig. 3b, the expression of *RRM1* and *RRM2* was significantly reduced (approximately 2-fold) in LPC028 and also in PANC-1 cells (Additional file 1: Figure S6) treated with perifosine versus untreated cells, while only minimal variations were observed for *hCNT1*, *hENT1*, *dCK*, and *CDA* expression. No significant changes were observed in the LPC006 cells (Fig. 3b). These results can at least in part explain the synergistic interaction of perifosine with gemcitabine in PDAC cells with high phospho-Akt expression.

### Perifosine and its combination with gemcitabine inhibit cell migration/invasion and upregulate the expression of E-cadherin

**Fig. 4:**
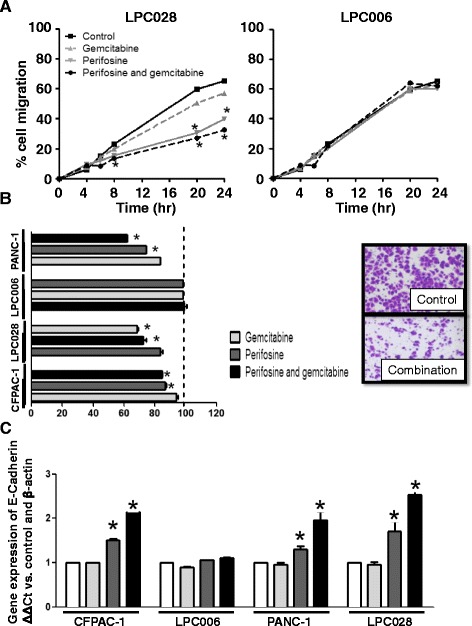
Effects of perifosine, gemcitabine and their combination on PDAC cells migration and invasion. **a** Results of wound-healing assay in LPC028 and LPC006 cells exposed to perifosine, gemcitabine or to their combination, at IC50 values for 24 h. **b** Results of invasion studies in the PDAC cells exposed for 24 h to perifosine, gemcitabine, or to their combination, at IC_50_ values (insert: representative pictures of LPC028 cells at 24 h, original magnification ×40). **c** Modulation of *E-cadherin* mRNA levels in LPC028, LPC006, PANC-1, and CFPAC-1 cells after 24-h exposure to perifosine, gemcitabine, or to their combination, at IC_50_ values, as determined by qRT-PCR. *Columns* or *points* mean values obtained from three independent experiments; *bars*, SEM. *Significantly different from controls

To determine the effects of perifosine, gemcitabine, and their combination on migratory behavior, a scratch mobility assay was performed in LPC028, LPC006 (Fig. 4a), CFPAC-1, and PANC-1 (Additional file 1: Figure S7). LPC028 showed a significant reduction of migration starting after 8 h exposure to perifosine with a reduction of the scratch-area of about 50%, and the perifosine/gemcitabine combination additionally reduced cell migration (*P* < 0.05; Fig. 4a left panel), while gemcitabine alone did not affect cell migration. No modulation of cell migration was observed in the LPC006 cells (Fig. 4a right panel). Similarly, the migration of these cells was not affected by MK-2206 alone and in combination with gemcitabine (Additional file 1: Figure S8).

LPC028, CFPAC-1, and PANC-1 cells treated with perifosine showed also a significantly reduced invasive potential, compared to untreated cells (Fig. 4b). In particular, the perifosine/gemcitabine combination was more effective in inhibiting invasion than perifosine-alone in LPC028 and PANC-1 cells, as shown by the significantly lower number of invading cells with Giemsa’s stain. However, no modulation of cell invasion was observed in the LPC006 cells.

Since previous studies suggested that the Akt signaling pathway suppressed E-cadherin expression [42], we investigated whether perifosine could affect the level of this target at both mRNA and protein level. Perifosine and its combination with gemcitabine significantly enhanced E-cadherin mRNA expression in LPC028, CFPAC-1, and PANC-1 (*P* < 0.05; Fig. 4c), while no changes were detected in LPC006 cells. Similarly, immunocytochemistry analysis in LPC028 cells illustrated a significant increase of E-cadherin protein staining after exposure to both perifosine and perifosine/gemcitabine combination (data not shown).

### Perifosine and its combination with gemcitabine affect cell cycle

Perifosine, gemcitabine and their combination affected cycle distribution of PDAC cells, as summarized in Additional file 2: Table S1. Perifosine significantly (P < 0.05) increased the percentages of LPC028 cells in S and G2/M phases (e.g., from 18.7 in the control to 26.1% in the S phase) after 72 h, while reducing the percentage of the cells in G0/G1. Similarly, the perifosine/gemcitabine combination significantly decreased the cells in G1 phase, while increasing the cells in S phase, up to 48.9%. Comparable perturbations of cell cycle were observed in the CFPAC-1 and PANC-1 cells, suggesting that perifosine might favor gemcitabine activity through a significant increase of cells in the S phase. Opposite modulation of cell cycle was observed in LPC006 cells, with only a slight increase of the cells in the G0/G1 phase and minimal modulations of the S and G2/M phase in cells exposed to perifosine/gemcitabine combination.

### Perifosine and its combination with gemcitabine enhance cell death and apoptosis

Analysis of the sub-G1 region of cell cycle perturbation demonstrated that the treatment with perifosine enhanced cell death (Additional file 2: Table S1). In particular, the LPC028 cells treated with the combination exhibited the largest sub-G1 signal (e.g., ≈20% in cells treated with perifosine/gemcitabine combination versus untreated cells).

**Fig. 5:**
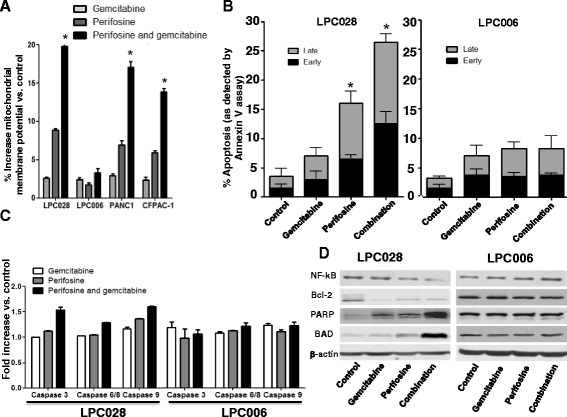
Apoptosis induction by perifosine, gemcitabine and their combination. **a** Mitochondrial membrane potential (as assessed by (DiOC) labelling) in LPC028, LPC006, PANC-1, and CFPAC-1 cells. **b** Annexin-V assay in LPC028 and LPC006 cells. **c** Modulation of caspase-3, caspase-6/-8/ and caspase-9 in LPC028 and LPC006 cells, as determined by a specific fluorometric assay described in the Methods section. **d** Representative Western blot pictures of apoptosis determinants in LPC006 and LPC028 cells. All these results refer to cells exposed for 24 h to perifosine, gemcitabine. or their combination at IC_50s_. *Columns*, mean values obtained from three independent experiments; *bars*, SEM. *Significantly different from controls

Moreover, we evaluated the variation of mitochondrial membrane potential in LPC028, LPC006, PANC-1, and CFPAC-1. As shown in Fig. 5a, the combination perifosine gemcitabine causes an increase of mitochondrial membrane potential in LPC028, PANC-1, and CFPAC-1 cells.

Further analysis of cell death by the Annexin-V/PI assay confirmed the induction of apoptosis by perifosine. Perifosine increased both early and late apoptosis, as shown in Fig. 5b (left panel) for the LPC028 cells. Moreover, the combination of perifosine and gemcitabine significantly increased the percentage of late apoptotic cells up to 26%. Similar results were observed in CFPAC-1 and PANC-1 cells (Additional file 1: Figure S9), whereas no apoptosis induction was detected in LPC006 cells (Fig. 5b right panel).

### Perifosine and its combination with gemcitabine activate caspases and pro-apoptotic factors, and downregulate Bcl-2 and NF-kB

In order to investigate the molecular mechanisms underlying apoptosis induction, we explored several potential cellular targets of perifosine, focusing on activation of the *initiator* caspases, caspase-8 and -9, and the *effector* caspases, caspase-3, and -6. Moreover, we studied the expression of various pro-apoptotic and anti-apoptotic proteins. As shown in Fig. 5c, perifosine and its combination with gemcitabine were able to increase the activity of caspase-3/-6/-8/-9 in LPC028 as well as CFPAC-1 and PANC-1 (Additional file 1: Figure S10) but not in the LPC006 cells, as determined by specific fluorometric caspase activity assays. However, Western blot analyses demonstrated the modulation of other important apoptotic markers. In particular, perifosine and perifosine/gemcitabine combination increased the expression of PARP and BAD, while reducing Bcl-2 and NF-kB expression in LPC028 cells. Conversely, none of these proteins was affected by the exposure to perifosine and its combination with gemcitabine in the LPC006 cells (Fig. 5d).

### Glut1 is overexpressed in the cells resistant to Akt inhibition, while its inhibition significantly reduces cell growth and induces apoptosis after gemcitabine/perifosine treatment

**Fig. 6:**
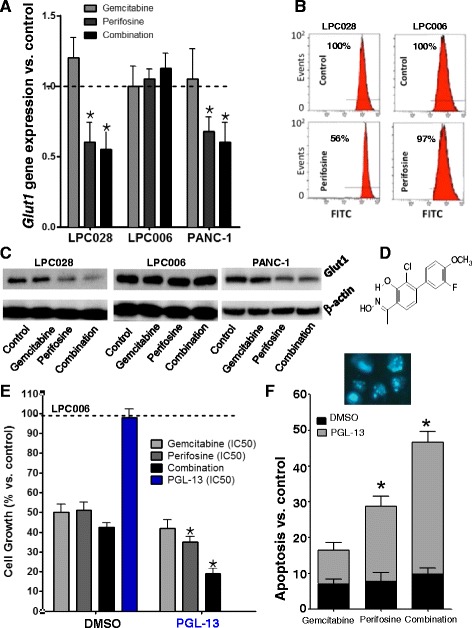
Role of Glut1 expression and inhibition in cell growth and apoptosis induction by perifosine, gemcitabine, and their combination. **a** Modulation of *Glut1* mRNA levels in LPC028, LPC006, and PANC-1 cells after 24-h exposure to perifosine, gemcitabine, or to their combination, at IC_50_ values, as determined by qRT-PCR. **b** Representative of Glut1 membrane-bound expression in LPC006 and LPC028 cells exposed for 24 h to perifosine at IC_50s_; **c** Representative Western blot pictures of Glut1 expression in LPC006, LPC028, and PANC-1 cells exposed for 24 h to perifosine, gemcitabine, or their combination at IC_50s_. **d** Structure of the compound PGL13. **e** Cell growth inhibition in LPC006 cells after 72-h exposure to perifosine, gemcitabine, or to their combination, at IC_50_ values, together with DMSO or with the Glut1 inhibitor PGL13, at 30 μM. **f** Apoptosis induction by perifosine, gemcitabine, and their combination as assessed by bisbenzimide staining as described in the Methods section (insert: representative pictures of apoptotic LPC006 cells after treatment with perifosine and gemcitabine, original magnification ×40). *Columns*, mean values obtained from three independent experiments; *bars*, SEM. *Significantly different from cells treated with DMSO

Since major oncogenic signaling pathways have been linked to increased glucose metabolism, and previous studies showed that stimulation of Akt1 induces Glut1 mRNA and protein accumulation, [43] we evaluated the expression of this key glucose transporter in the LPC028 and LPC006 cells. As shown in the Fig. 6a, *Glut1* mRNA levels were significantly reduced after treatment with perifosine alone and in combination with gemcitabine in the LPC028 and PANC-1 cells, whereas no modulation was detected in the LPC006 cells. However, since PI3K/AKT/mTOR signaling seems to play an essential role in trafficking of Glut1 from recycling endosomes and/or retention of Glut1 at the plasma membrane [44], we performed further studies to evaluate the amount of membrane-bound Glut1 with FACS analysis (Fig. 6b). In the LPC028 cells, we observed a significant reduction (*P* < 0.05) of the membrane-bound expression of Glut1 after treatment with perifosine (56% compared to untreated cells). Further studies with Western blot clearly demonstrated the overexpression of Glut1 in the LPC006 compared to the LPC028 and PANC-1 cells. A high expression of Glut-1 was also observed in PANC-1 cells (Fig. 6c). Moreover, Glut1 expression was not reduced by Akt inhibition (Fig. 6c). We therefore investigated whether inhibition of Glut1 by the novel specific compound PGL13 (Fig. 6d) can at least in part overcome the inherent resistance of the LPC006 cells to perifosine and other Akt inhibitors. Remarkably, the Glut1 inhibitor alone caused only a slight reduction of cell growth (<10%), but its combination with perifosine reduced significantly the percentage of surviving cells compared to perifosine alone (Fig. 6e). Furthermore, the combination of PGL13 with both perifosine and gemcitabine led to a more dramatic drop in the number of surviving cells, up to −81%, compared to control which was associated with strong apoptosis induction, as detected by characteristic apoptotic nuclear morphological features with fluorescence microscopy. In particular the LPC006 cells exposed to PGL13 with both perifosine and gemcitabine had an apoptotic index of 27%, which was similar to the apoptotic index of the LPC028 cells treated with perifosine and gemcitabine (Fig. 6f). The effect of a combined Akt inhibitor/anti-Glut1 treatment was further tested with MK-2205 and NVP-BEZ235, where it led to a −14% and −20% decrease in cell viability compared with these drugs alone (Additional file 1: Figure S11). Thus, inhibition of Glut1 promoted anti-Akt-mediated cell death, and this combined treatment shows promise for future investigation in the treatment of PDAC.

## Discussion

The present study supports a role for phospho-Akt as a prognostic factor in PDAC patients, and unravels its potential role as a target for the synergistic interaction of anti-Akt agents and gemcitabine through modulation of apoptotic and invasive processes.

Several studies demonstrated that PDAC tissues have increased activation of the PI3K/Akt, as assessed with the phosphorylation of Akt, and this has been associated with higher histological tumor grade [45] and worse prognosis [46, 47]. In the present study, we further explored the clinical relevance of phospho-Akt by screening its expression in a homogeneous cohort of 100 surgically resected PDACs. In agreement with the previous studies, even considering several clinicopathological parameters, phospho-Akt expression was the only factor correlated to differential clinical outcome. However, a systematic review and meta-analysis of prognostic tissue biomarkers for PDAC, including phospho-Akt among the 22 markers associated with limitless replicative potential eligible for examination, showed that only Ki-67 maintained statistically significant associations with outcome [48]. These discrepancies might be attributed to the different experimental procedures used, including antigen retrieval technique, antibody characteristics, and dilution, as well as observer variability in staining pattern description and cutoff point selection. Therefore, in the present study we have chosen an antibody that was previously validated in an immunohistochemical study on 102 colorectal cancer FFPE samples [49] and we have used image analysis software to calculate expression as a continuous parameter, in order to facilitate the identification of cutoff points. This method allowed the assignment of the specimens to different categories, including a subset of tissues (about 14%) characterized by extremely high expression of phospho-akt, which could clearly influence the prognostic value. Indeed this cutoff point identified a group of patients with very poor outcome, who should be treated with more aggressive, novel therapeutic approaches.

Of note, recent genomic studies showed that the PI3K/Akt signaling is among the core signaling pathways leading the intrinsic aggressiveness of PDAC, suggesting that in the *PDAC actionable genome* about 9 and 6% of the cases are Akt- and PI3K-dependent, respectively [50]. These data underline the potential importance of specific inhibitors of PI3K/Akt as novel effective therapeutics in a selected subpopulation of PDAC patients. Moreover, activation of this signaling pathway is associated with PDAC chemoresistance [13, 51], supporting the hypothesis that Akt inhibitors might also be used to overcome resistance towards conventional cytotoxic agents.

Several Akt/PI3K inhibitors are being developed. The first generation of these inhibitors includes LY294002 and wortmannin, which were tested to elucidate the value of Akt/PI3K as therapeutic target [52]. However, due to the unfavorable pharmaceutical properties, toxicity, and crossover inhibition of other lipid and protein kinases, these compounds were not used in clinical studies [51].

More recently, several small molecules that inhibit the PI3K/Akt signaling entered clinical development, but more information on their activity in the preclinical setting is warranted. For instance, a recent study showed the potential inhibition of autophagy by perifosine demonstrating that this drug impairs the autophagic flux in HepG2 and U87 MG cells, which is related to defects in intracellular cholesterol transport [53]. These results might be relevant for PDAC because some research lines point at autophagy as a tumor-promoting mechanism. Although a better understanding of the complexity of autophagy is needed, the modulation of this process might therefore open new opportunities for the therapeutic use of autophagy inhibitors [54]. Further research to identify the precise mechanisms of autophagy maturation may therefore provide a new insight into the antiproliferative action of perifosine.

Our results demonstrate that perifosine is the targeted anti-PI3K/Akt antitumor agent demonstrating the most potent growth inhibitory effects in a panel of human PDAC cells characterized by distinct molecular properties. Limited published preclinical research focusing on this issue in PDAC reported similar cytotoxic activity of perifosine in PANC-1, MIA PaCa-2, and AsPC-1 cells [55]. Sensitivity to perifosine in the PDAC cells also fell within the range of IC_50_ values previously reported in PDAC cell lines and spheroids for other Akt inhibitors, such as NVP-BEZ-235 [56, 57].

Furthermore, perifosine interacted synergistically with gemcitabine in PDAC cells with high phospho-Akt expression, but antagonistic in cells with low phospho-Akt expression. Synergism was associated with inhibition of migration/invasion and induction of apoptosis. These results are in agreement with previous studies showing synergistic interaction of gemcitabine with perifosine in PANC-1 cells and xenografts [55] as well as enhanced apoptotic cell death after combined treatment with paclitaxel in chemoresistant ovarian cancer cells [58].

However, most previous studies were performed in ATCC cell lines, which showed similar results [59], while, to more effectively develop targeted compounds, it will be helpful to understand why these agents fail when they do. Thus, in the present study, cell growth inhibitory effects of perifosine, gemcitabine, and their combination were evaluated in several representative PDAC cells, including primary PDAC cell cultures. For the LPC028 model, we demonstrated that perifosine inhibited cell growth, both in monolayer cell cultures and in cells growing as spheroids, whereas LPC006 cells and spheroids were not affected. Similarly, the perifosine/gemcitabine combination had synergistic effects only in the cells with high phospho-Akt or intermediate/high values of *Akt1* mRNA, as determined by RT-PCR. Conversely, this combination was antagonistic in the cells with low *Akt1*, and phospho-Akt1 expression. An important limitation of our findings is the use of a single-cell culture (LPC006) as a model of low phospho-Akt1. However, the results in two PDAC models (LPC028 and PANC-1) with high phospho-Akt1 levels were similar. These data suggest that the expression and activation of Akt might therefore be used to tailor perifosine therapy.

Importantly, two specific ELISA for the Akt Ser473 and the Thr308 phosphorylation showed that perifosine effectively reached and inhibited its targets in the LPC028 and PANC-1 cells, and the combination with gemcitabine additionally inhibited Akt activation in these cells. The present study demonstrated also that perifosine interfered with pivotal determinants for the activity of gemcitabine. In particular, we observed that perifosine and its combination with gemcitabine significantly reduced the expression of RRM1 and RRM2 in the cells with a high expression of Akt, while this effect was not statistically significant in the cells with low Akt expression. RR is a key target of gemcitabine activity and previous studies correlated the expression of its subunits to gemcitabine sensitivity in PDAC cells [60, 61]. Therefore, the synergistic interaction between perifosine and gemcitabine might be explained, at least in part, by the modulation of gemcitabine sensitivity through RRM1 and RRM2 suppressions.

**Fig. 7:**
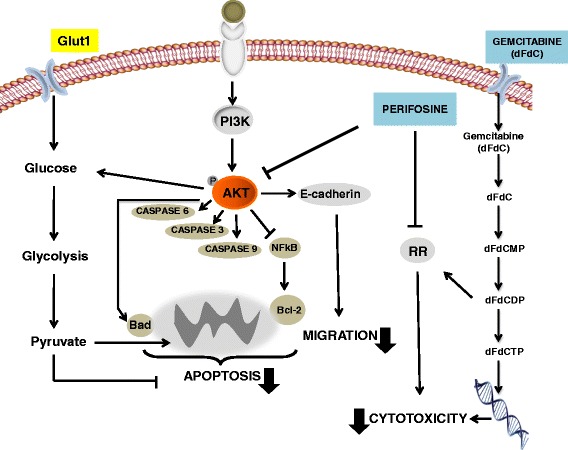
Molecular mechanisms involved in the synergistic interaction of perifosine with gemcitabine. The main upstream activator of Akt is phosphatidylinositol-3 kinase (PI3K), which is activated in the response to a variety of growth stimuli through receptor tyrosine kinases and G protein-coupled receptors. This kinase phosphorylates phosphatidylinositol-4,5-diphosphate (PIP2), which results in generation of phosphatidylinositol-3,4,5-triphosphate (PIP3). PIP3 interacts with the pleckstrin homology (PH) domain of Akt, leading to translocation of Akt to the cell membrane, and phosphorylation at Thr308 and Ser473. Perifosine inhibits Akt activation and enhances the growth inhibitory effects of gemcitabine through its pronounced pro-apoptotic, anti-invasive effects, as well as by inhibiting the cell proliferation, followed by modulation of ribonucleotide reductase (RR), potentially facilitating gemcitabine cytotoxicity. Moreover, Akt inhibition reduce Glut1 activity reducing glucose influx and thereby favouring apoptosis induction and sensitizing PDAc cells to treatment with cytotoxic agents

However, our results suggested that the synergistic interaction of perifosine with gemcitabine is associated with other important molecular mechanisms affecting PDAC aggressiveness (Fig. 7). In agreement with previous observations showing the reduction of cell migration/invasion through Akt inhibition [16, 62], we observed that perifosine and its combination with gemcitabine markedly reduced cell migration and invasion in PDAC cells. Several classes of proteins are involved in this invasive behavior, including cell-cell adhesion molecules like members of immunoglobulin and calcium-dependent cadherin families and integrins. In line with previous evidence on inverse relationship between Akt and E-cadherin expression [42], we demonstrated that perifosine increased the expression of E-cadherin in LPC028, CFPAC-1, and PANC-1 cells. This can at least in part explain our findings on the reduction of migration determined by perifosine. Furthermore, Toll et al. [63] showed that decreased E-cadherin was associated with poor prognosis of PDAC patients, supporting the studies on novel compound which can modulate the expression of this protein.

Since the Akt signaling pathway plays an important role in cell survival process, its blockage can result in activation of programmed cell death [15]. Thus, we further evaluated the effect of perifosine on cell cycle perturbation and apoptosis induction. Previous findings on the effect of perifosine after 24 h treatment showed induction of G_2_/M arrest, potentially favoring the activity of 6-thioguanine [64]. Our results showed that after 72 h, perifosine treatment was associated with an increase in the percentage of cells in the G_0_/G_1_, and S phase, potentially favoring the cytotoxic activity of gemcitabine. This modulation of the cell cycle was associated with significant induction of apoptosis, as determined by multiple methods, such as analysis of sub-G1, mitochondria membrane potential and Annexin-V/PI. In order to investigate the mechanisms underlying the activation of programmed cell death, we checked the modulation of critical factors involved in the apoptotic cascades. Previous studies showed that drug-induced Akt deactivation was associated with activation of pro-apoptotic factors, including caspase-9 and BAD, as well as with a parallel decrease in the expression of the anti-apoptotic factors Bcl-2 and NF-kB [22, 65]. Our studies showed similar results after exposure of the PDAC cells to perifosine.

Despite increasing evidence on the pivotal role of PI3K/Akt signaling in cancer, the strategies to hit PI3K/Akt/mTOR pathway have failed to demonstrate therapeutic activity in most ongoing clinical trials, and a previous phase II study testing perifosine in previously untreated patients with locally advanced, unresectable, or metastatic PDAC, was terminated as a result of unacceptable adverse events [66].

It is already known that in PDAC cells, dual PI3K-mTOR inhibition induces rapid overactivation of MAPK pathway through a PI3K-independent pathway [67], and that drug resistance may be overcome by inhibition of parallel oncogenic-dependent pathways, such as with the dual MEK and PI3K/mTOR blockade [57].

One strategy to overcome resistance consists into identifying key molecular differences in the tumors that are less likely to respond. Oncogenic *KRAS* drives metabolic reprogramming in tumor cells by increasing aerobic glycolysis, and recent studies showed that subtypes of PDAC cells with distinct metabolite levels associated with glycolysis, lipogenesis, and redox pathways, confirmed at the transcriptional level. The glycolytic and lipogenic subtypes showed striking differences in glucose and glutamine utilization, as well as mitochondrial function, and corresponded to differences in cell sensitivity to inhibitors of glycolysis, glutamine metabolism, lipid synthesis, and redox balance [68]. In the present study we demonstrated that the resistant LPC006 cells were characterized by overexpression of Glut1. Remarkably, the inhibition of Glut1 dramatically enhanced perifosine and perifosine/gemcitabine-induced cell death, suggesting a cooperativity between Akt inhibitors and Glut1 inhibition. Agents directly inhibiting Glut1 are in early phase evaluations, and a few preclinical studies have demonstrated that Glut inhibitors led to diminish tumor growth in vitro and in vivo [69]. However, the altered expression of Glut1 might also influence the sensitivity of tumor cells to chemotherapy, since a recent study showed that the knockdown of *Glut1* sensitizes head and neck cancer cells to the chemotherapy drug cisplatin [70]. To our knowledge, this is the first study showing that Glut1 inhibitors can restore the repression of aerobic glycolysis induced by PI3K/mTOR inhibitors in resistant cells, and favor their synergistic interaction with cytotoxic compounds. These results should prompt further studies to understand how PDAC cell metabolism might affect sensitivity to new anti-signaling therapies and to identify promising therapeutic targets that might be exploited by combination therapies.

## Conclusions

Our data support the analysis of phospho-Akt expression as both a prognostic and a predictive biomarker, for the rational development of novel therapies targeting the Akt pathway in PDAC. In particular, we observed that phospho-Akt expression levels influence the antitumor activity of perifosine, as well as the synergistic interaction with gemcitabine, through its ability to attack key mechanisms involved in the proliferation, cell cycle control, apoptosis and migration/invasion properties. Finally, we demonstrated that inhibition of Glut1 overcame resistance to this combination treatment and might provide the basis for the development of new therapeutic approaches with Akt inhibitors in patients with PDAC.

## Additional files

Additional file 1: Figure S1. PFS curves according to phospho-Akt expression in radically-resected PDACs, showing that patients with high and “very high” phospho-Akt (right panel) had a significantly worse PFS. **Figure S2.** Growth inhibitory effects after MK-2206 exposure in LPC006 (72-hours). **Figure S3.** Growth inhibitory effects after 72 hours exposure to perifosine, gemcitabine or their combination at a fixed ratio based on IC50 values in CFPAC-1 and PANC-1 cells. On the X-axis the drug concentrations for the combination are referred to gemcitabine. **Figure S4.** Phospho-Akt (serine residue-473) expression, normalized to total Akt, after 4-hour exposure, as determined by ELISA. **Figure S5.** **A** Phospho-Akt (serine residue-473) expression, normalized to total Akt, after 24-hour exposure. **B** Phospho-Akt (threonine residue-308) expression, normalized to total Akt, after 24-hour exposure, as determined by ELISA. **Figure S6.** Expression of gemcitabine determinants in PANC-1 cells treated with perifosine, as determined by qRT-PCR. Dashed line, values in untreated samples. **Figure S7.** Wound-healing assay in CFPAC-1 and PANC-1 exposed to perifosine, gemcitabine or their combination (IC50 values, 24 hours). **Figure S8.** Wound-healing assay in LPC006 exposed to MK-2206, gemcitabine or to their combination (IC50 values, 24 hours). **Figure S9.** Annexin-V assay in LPC028 and LPC006. **Figure S10**: Modulation of caspase-3, caspase-6/-8/ and caspase-9 in CFPAC-1 and PANC-1, as determined by a specific fluorometric assay. **Figure S11.** Cell growth inhibition in LPC006 cells after 72-hour exposure to MK-2205, NVP-BEZ235 at IC50 values, together with DMSO or with the Glut1 inhibitor PGL13, at 30 μM. *Points*, or *Columns*, mean values obtained from three independent experiments; bars, SEM. *Significantly different from controls. (PPTX 328 kb)
Additional file 2:
**Table S1.** Effects of gemcitabine and perifosine and their combination on cell cycle distribution and on cell death (sub-G1). (DOCX 14 kb)
