# Supplementary material for: Phospho-Akt overexpression is prognostic and can be used to tailor the synergistic interaction of Akt inhibitors with gemcitabine in pancreatic cancer
Source: J Hematol Oncol. 2017 Jan 6;10:9. doi: 10.1186/s13045-016-0371-1 (PMC5219723; doi:10.1186/s13045-016-0371-1)
Supplement: Additional file 1: Figure S1. — PFS curves according to phospho-Akt expression in radically-resected PDACs, showing that patients with high and “very high” phospho-Akt (right panel) had a significantly worse PFS. Figure S2. Growth inhibitory effects after MK-2206 exposure in LPC006 (72-hours). Figure S3. Growth inhibitory effects after 72 hours exposure to perifosine, gemcitabine or their combination at a fixed ratio based on IC50 values in CFPAC-1 and PANC-1 cells. On the X-axis the drug concentrations for the combination are referred to gemcitabine. Figure S4. Phospho-Akt (serine residue-473) expression, normalized to total Akt, after 4-hour exposure, as determined by ELISA. Figure S5. A Phospho-Akt (serine residue-473) expression, normalized to total Akt, after 24-hour exposure. B Phospho-Akt (threonine residue-308) expression, normalized to total Akt, after 24-hour exposure, as determined by ELISA. Figure S6. Expression of gemcitabine determinants in PANC-1 cells treated with perifosine, as determined by qRT-PCR. Dashed line, values in untreated samples. Figure S7. Wound-healing assay in CFPAC-1 and PANC-1 exposed to perifosine, gemcitabine or their combination (IC50 values, 24 hours). Figure S8. Wound-healing assay in LPC006 exposed to MK-2206, gemcitabine or to their combination (IC50 values, 24 hours). Figure S9. Annexin-V assay in LPC028 and LPC006. Figure S10: Modulation of caspase-3, caspase-6/-8/ and caspase-9 in CFPAC-1 and PANC-1, as determined by a specific fluorometric assay. Figure S11. Cell growth inhibition in LPC006 cells after 72-hour exposure to MK-2205, NVP-BEZ235 at IC50 values, together with DMSO or with the Glut1 inhibitor PGL13, at 30 μM. Points, or Columns, mean values obtained from three independent experiments; bars, SEM. *Significantly different from controls. (PPTX 328 kb) [file 13045_2016_371_MOESM1_ESM.pptx]

## Slide 1
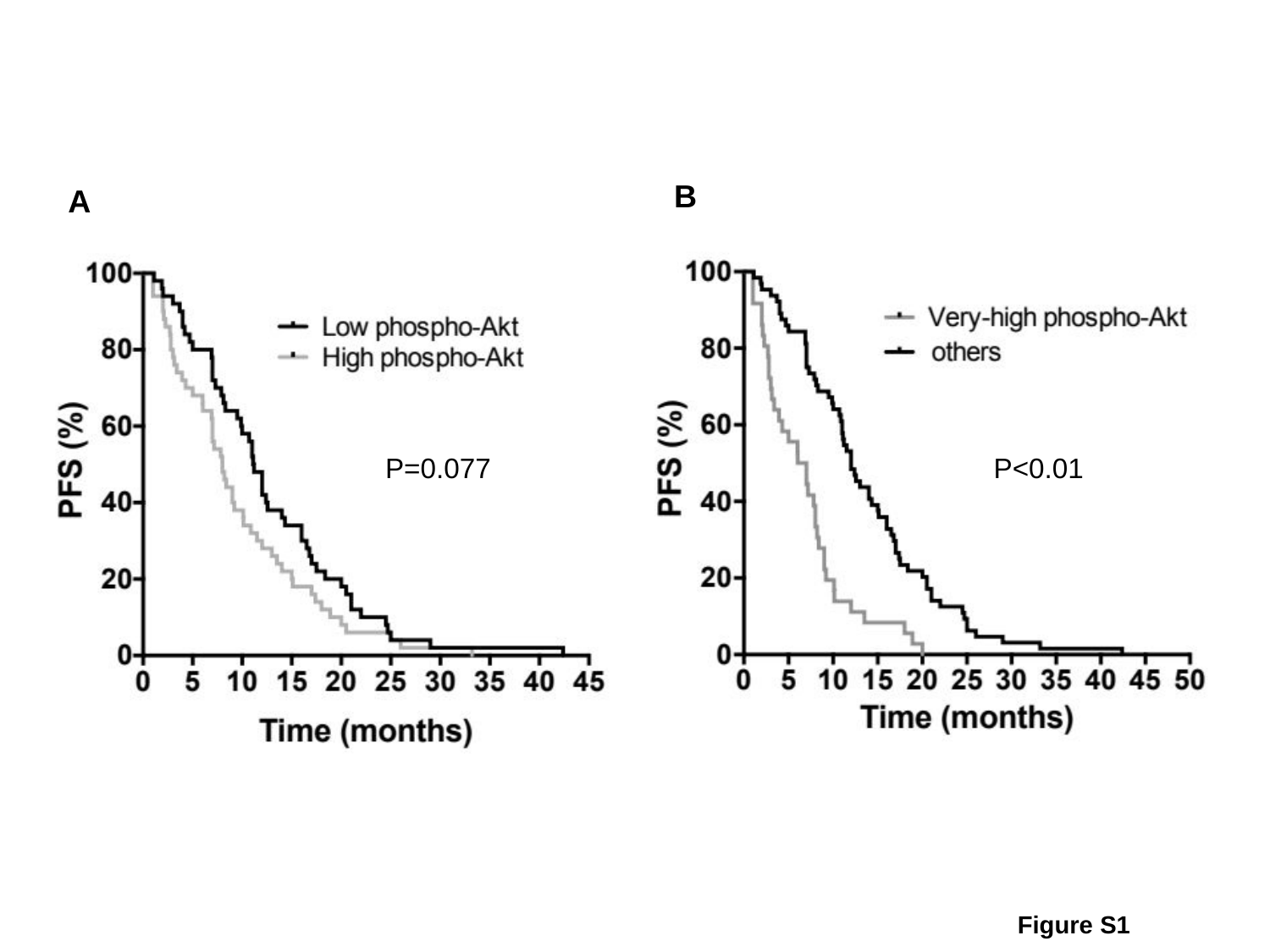

B
A
P=0.077
P<0.01
Figure S1

## Slide 2
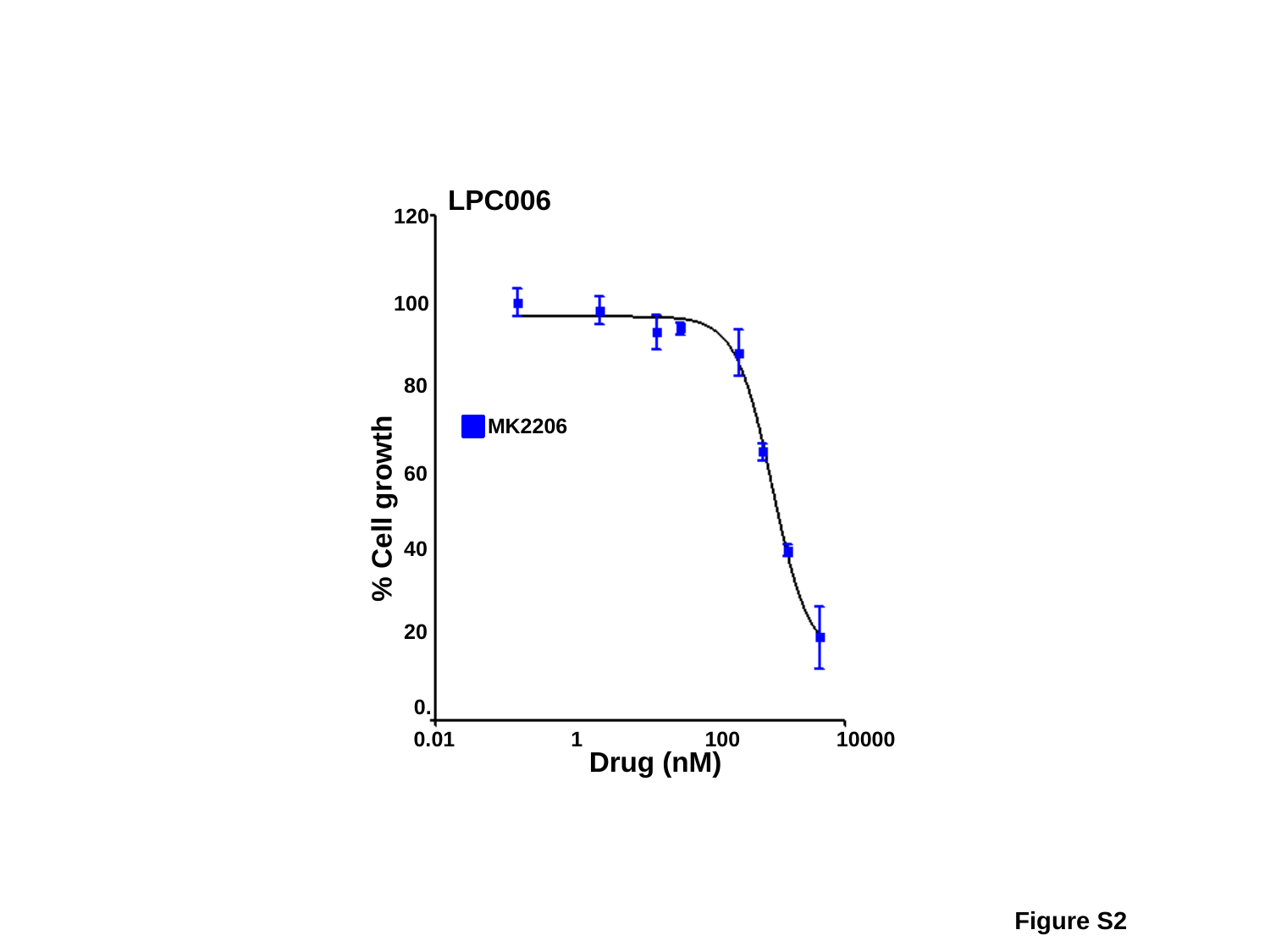

LPC006
120
100
80
60
40
20
0.
% Cell growth
MK2206
0.01
1
100
10000
0.01
1
100
10000
Drug (nM)
Figure S2

## Slide 3
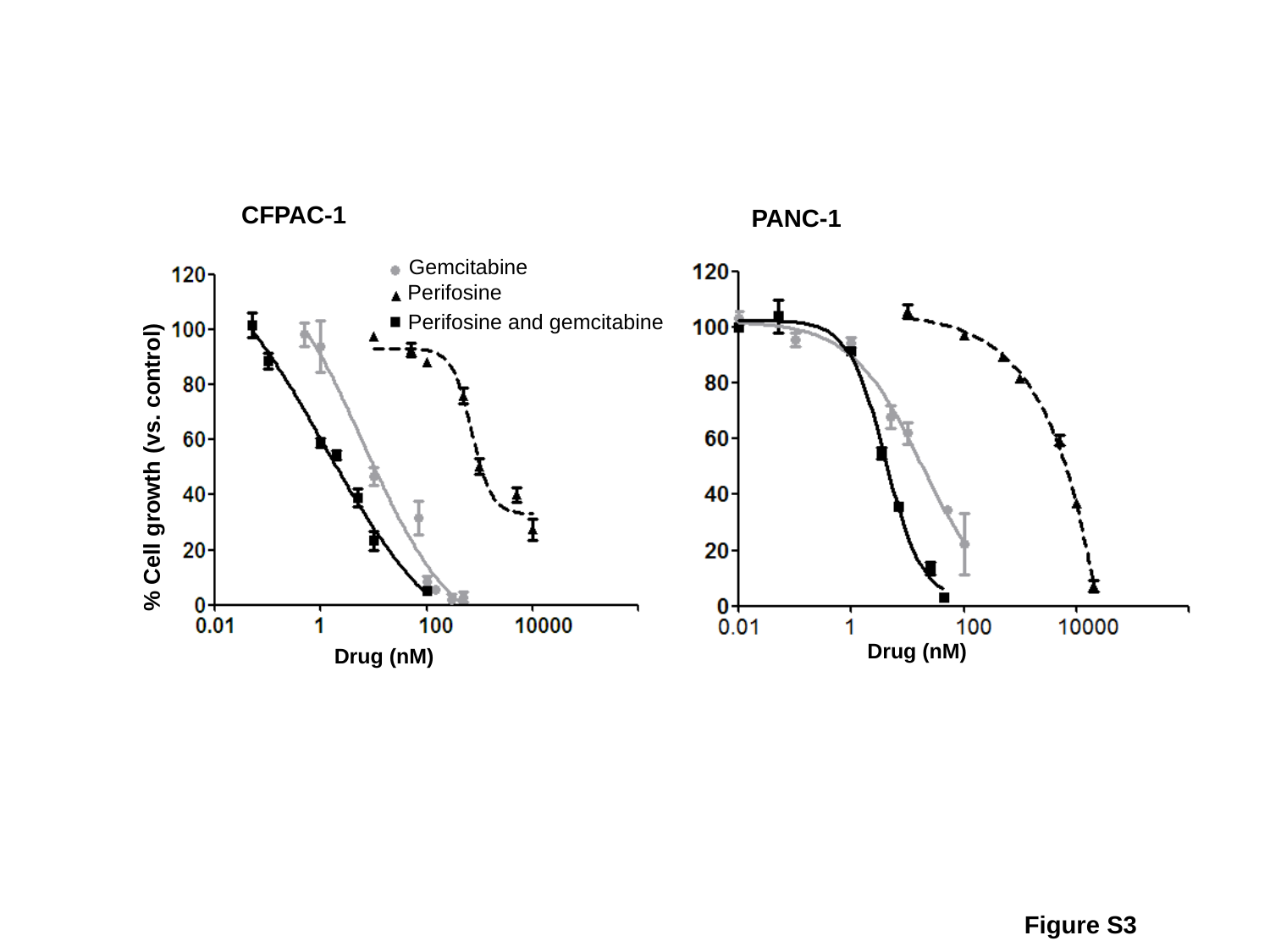

CFPAC-1
PANC-1
 Gemcitabine
% Cell growth (vs. control)
Drug (nM)
Perifosine
Perifosine and gemcitabine
Drug (nM)
Figure S3

## Slide 4
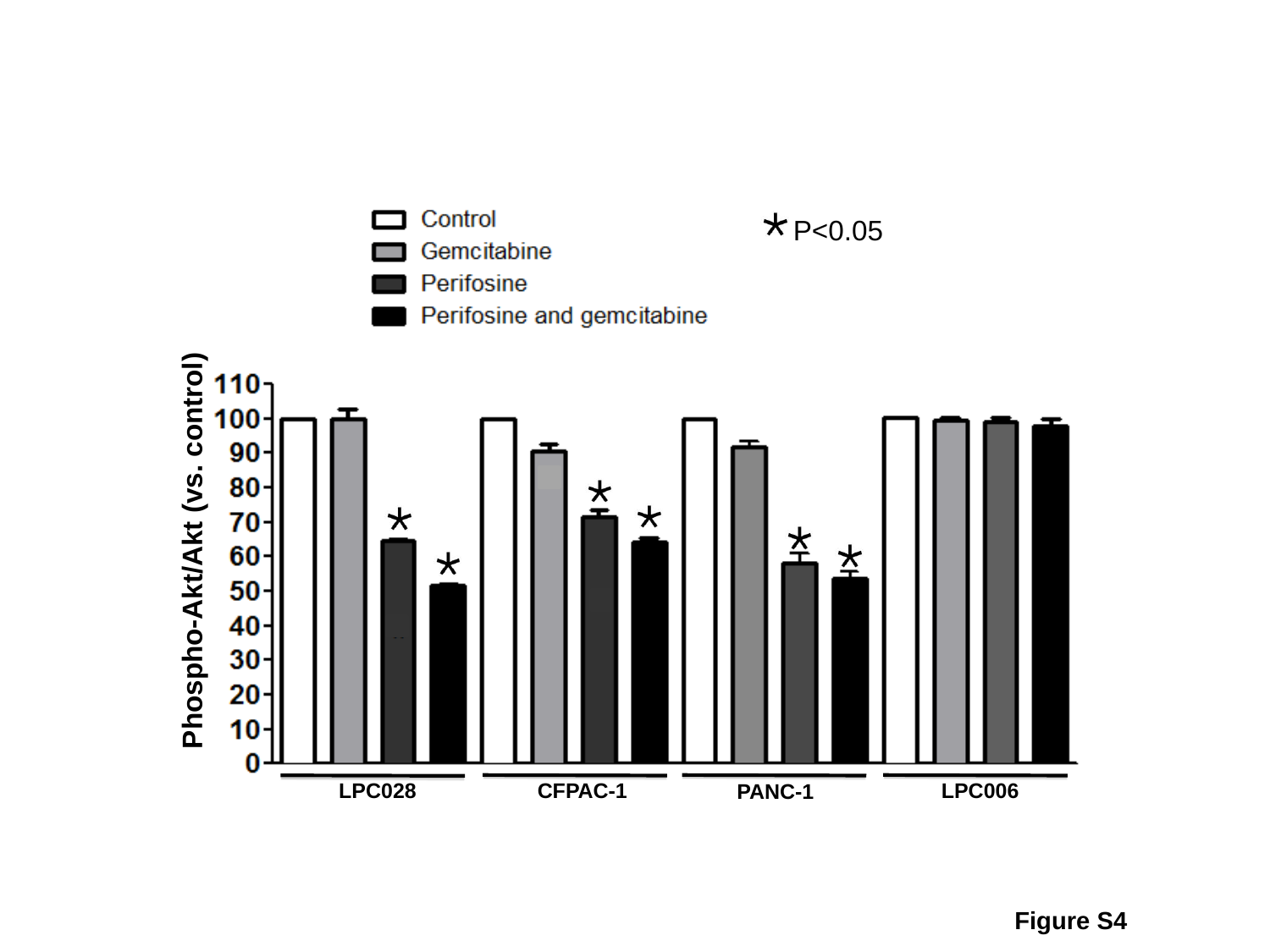

Phospho-Akt/Akt (vs. control)
CFPAC-1
LPC006
LPC028
PANC-1
P<0.05
Figure S4

## Slide 5
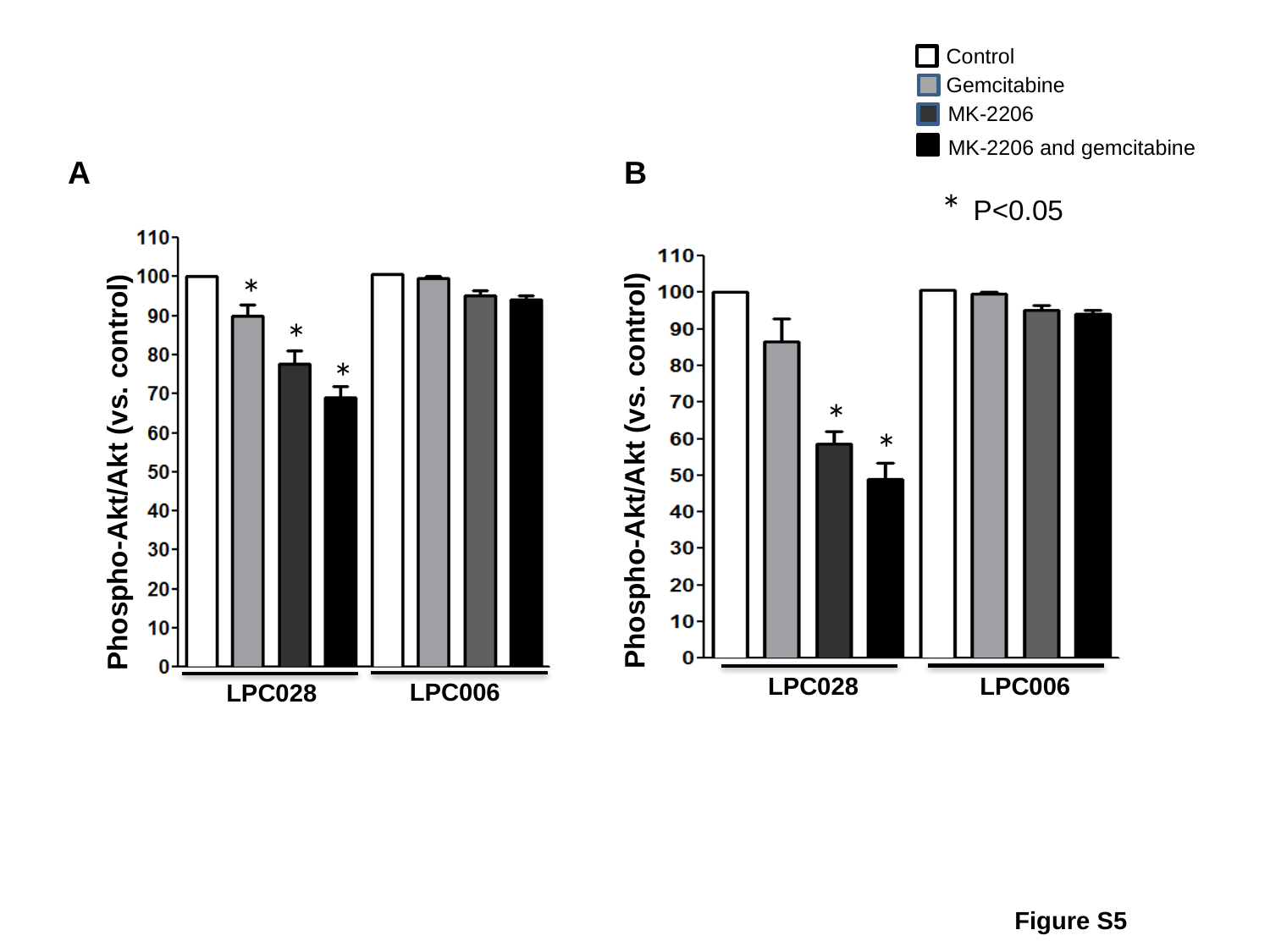

Control
Gemcitabine
MK-2206
MK-2206 and gemcitabine
B
A
*
P<0.05
LPC006
LPC028
*
*
*
*
*
Phospho-Akt/Akt (vs. control)
Phospho-Akt/Akt (vs. control)
LPC006
LPC028
Figure S5

## Slide 6
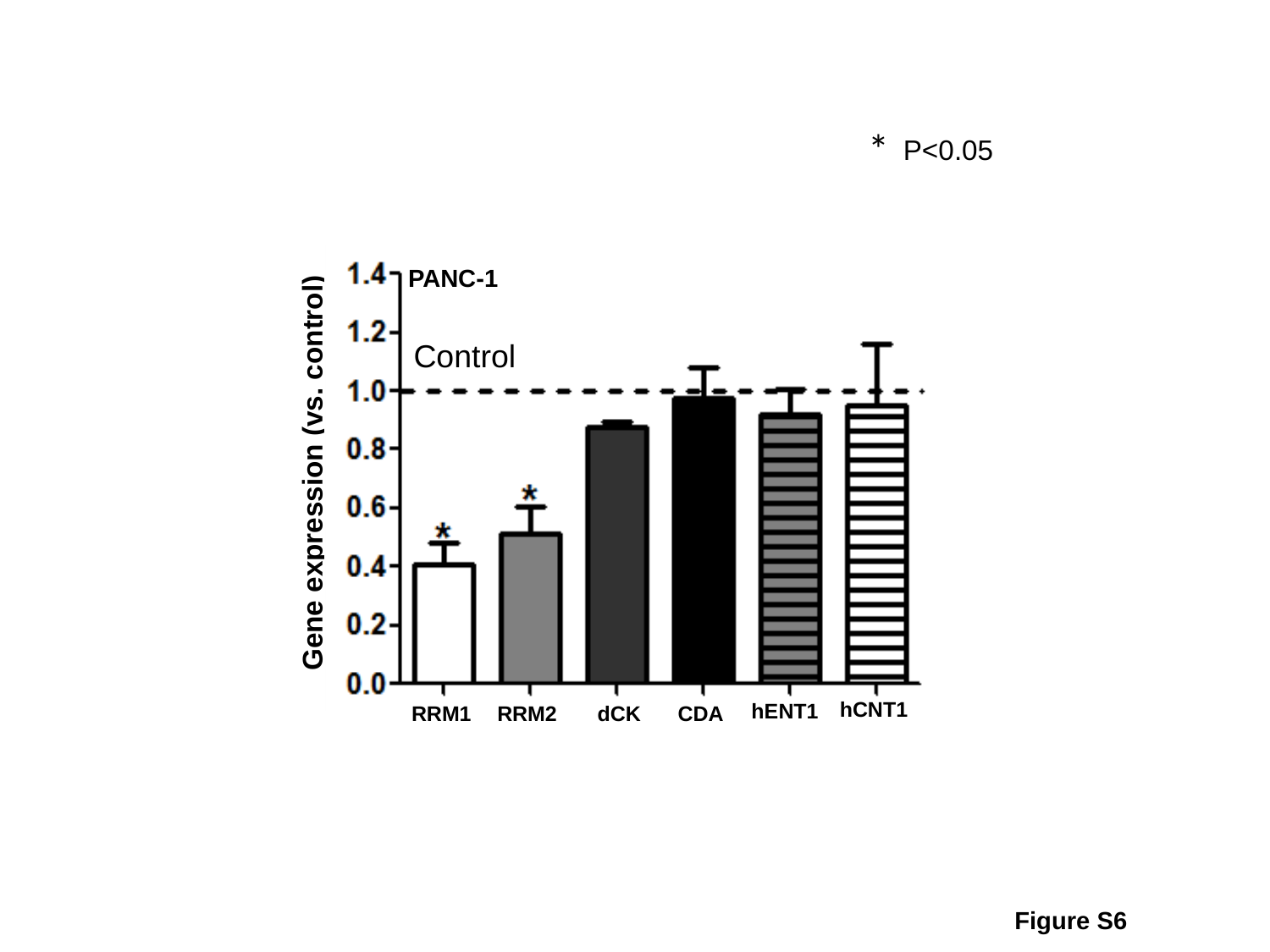

*
P<0.05
PANC-1
Control
hCNT1
hENT1
RRM1
RRM2
dCK
CDA
Gene expression (vs. control)
Figure S6

## Slide 7
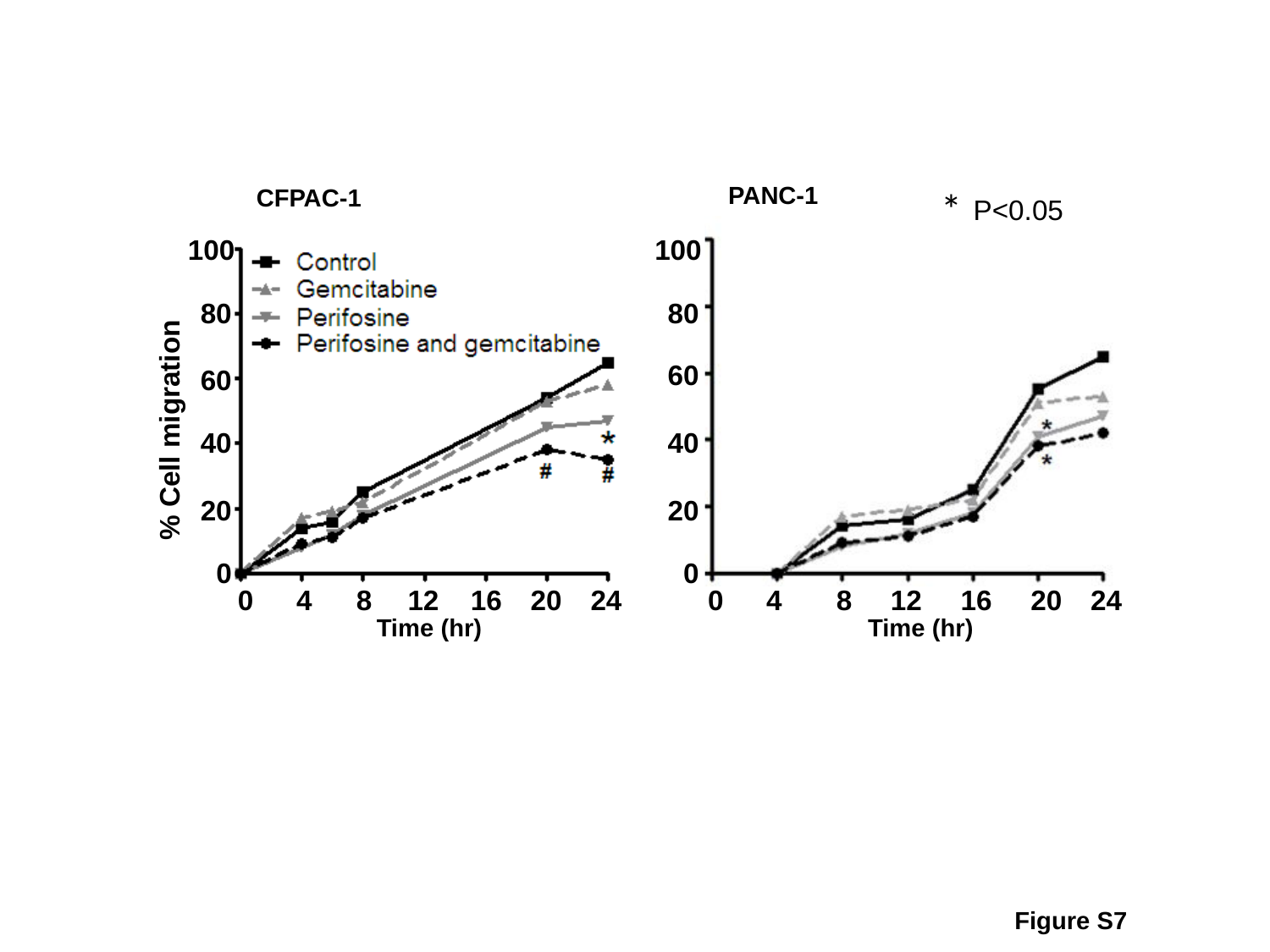

PANC-1
CFPAC-1
% Cell migration
Time (hr)
100
80
60
40
20
0
*
P<0.05
100
80
60
40
20
0
0
4
8
12
16
20
24
0
4
8
12
16
20
24
Time (hr)
Figure S7

## Slide 8
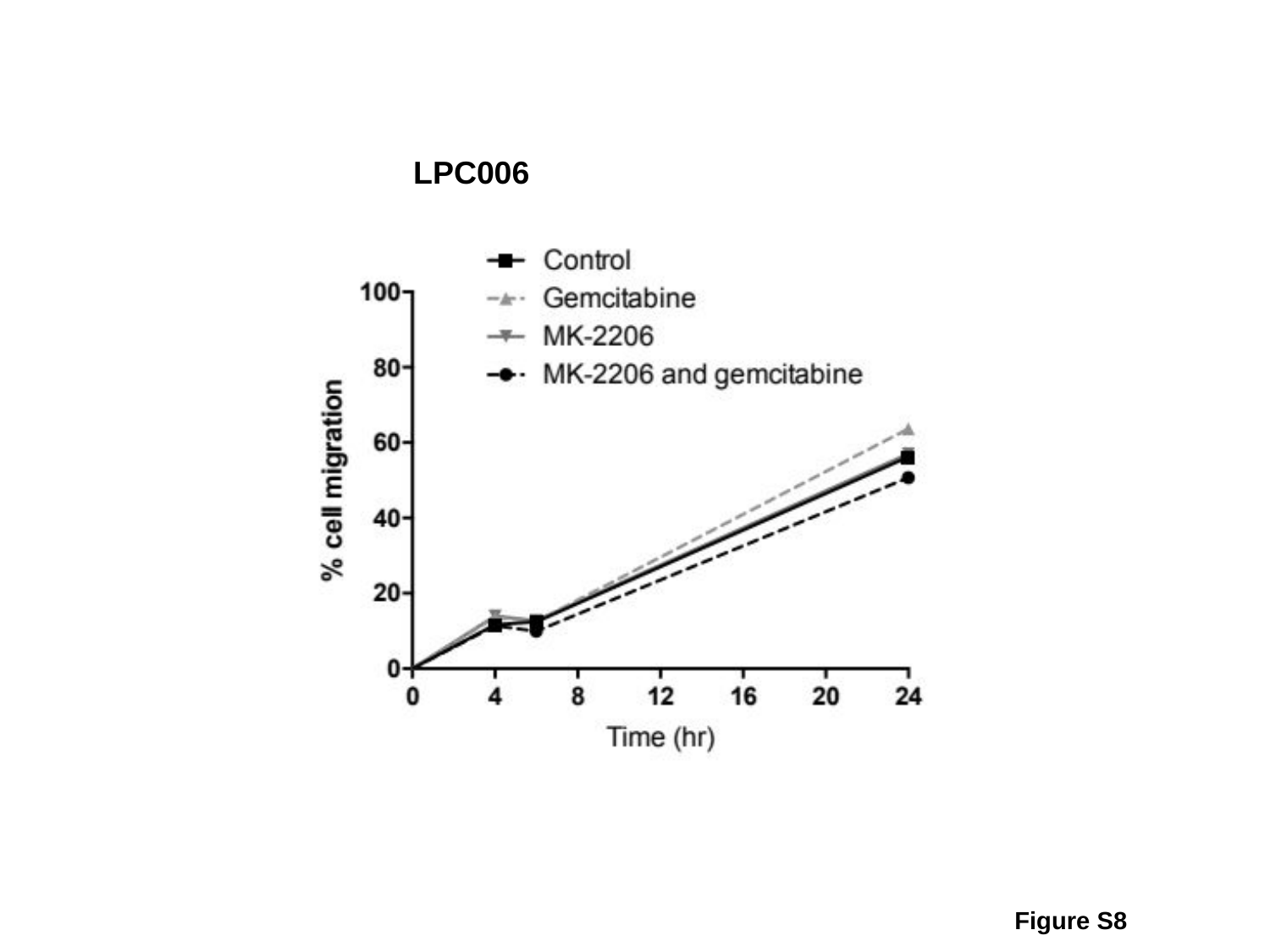

LPC006
Figure S8

## Slide 9
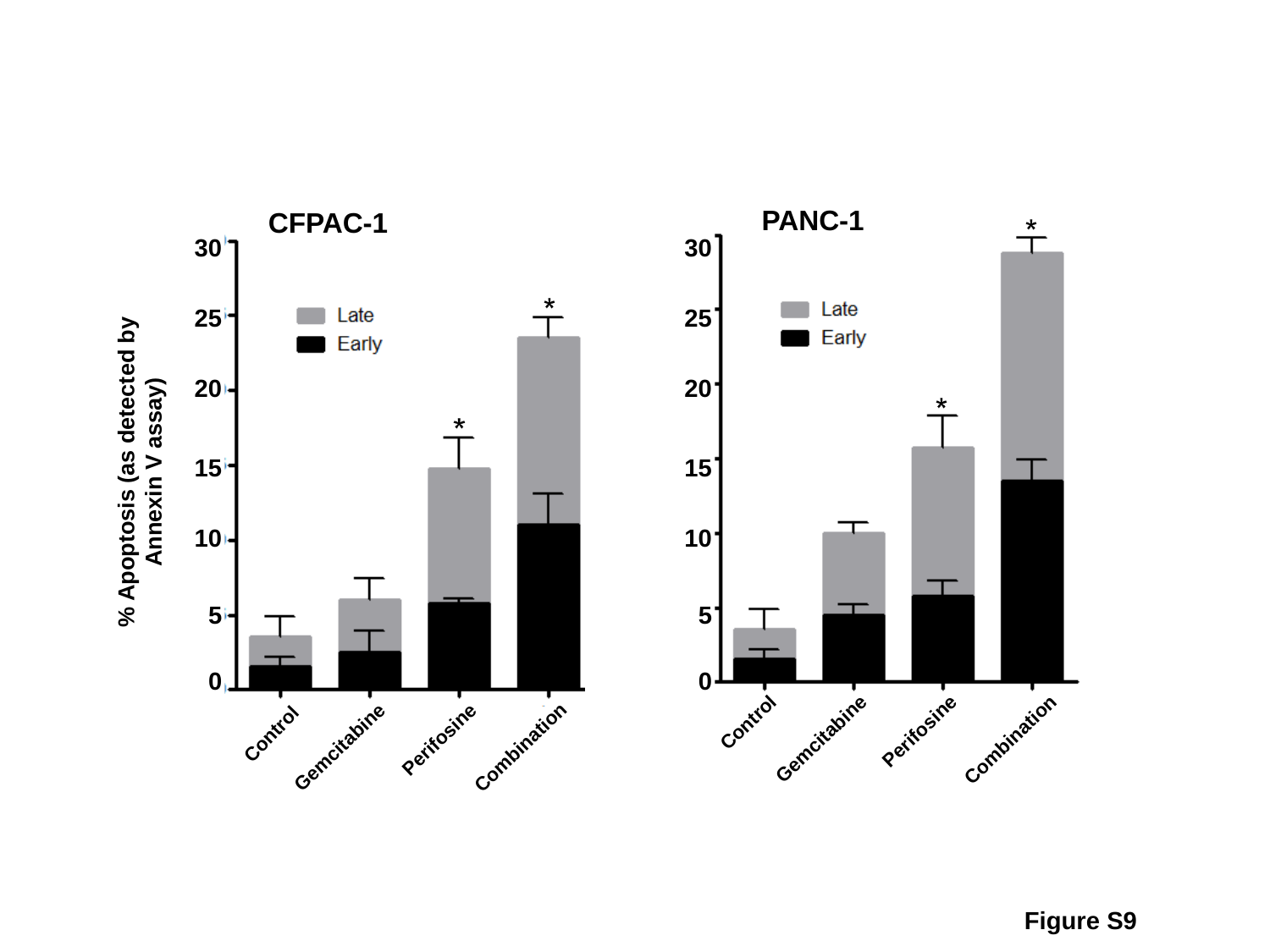

PANC-1
CFPAC-1
30
25
20
15
10
5
0
30
25
20
15
10
5
0
% Apoptosis (as detected by Annexin V assay)
Control
Perifosine
Control
Gemcitabine
Perifosine
Combination
Gemcitabine
Combination
Figure S9

## Slide 10
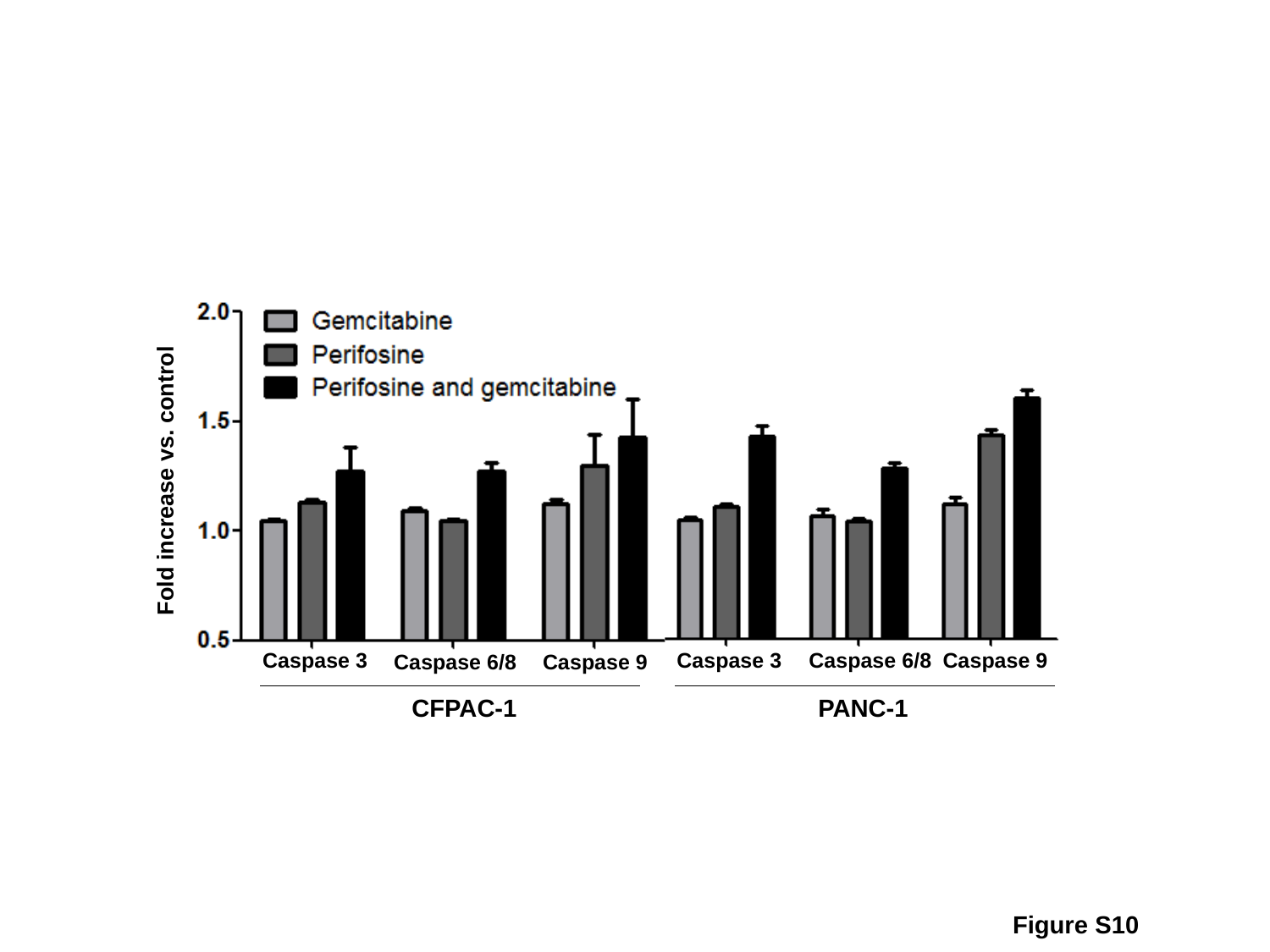

Fold increase vs. control
Caspase 3
Caspase 3
Caspase 6/8
Caspase 9
Caspase 6/8
Caspase 9
CFPAC-1
PANC-1
Figure S10

## Slide 11
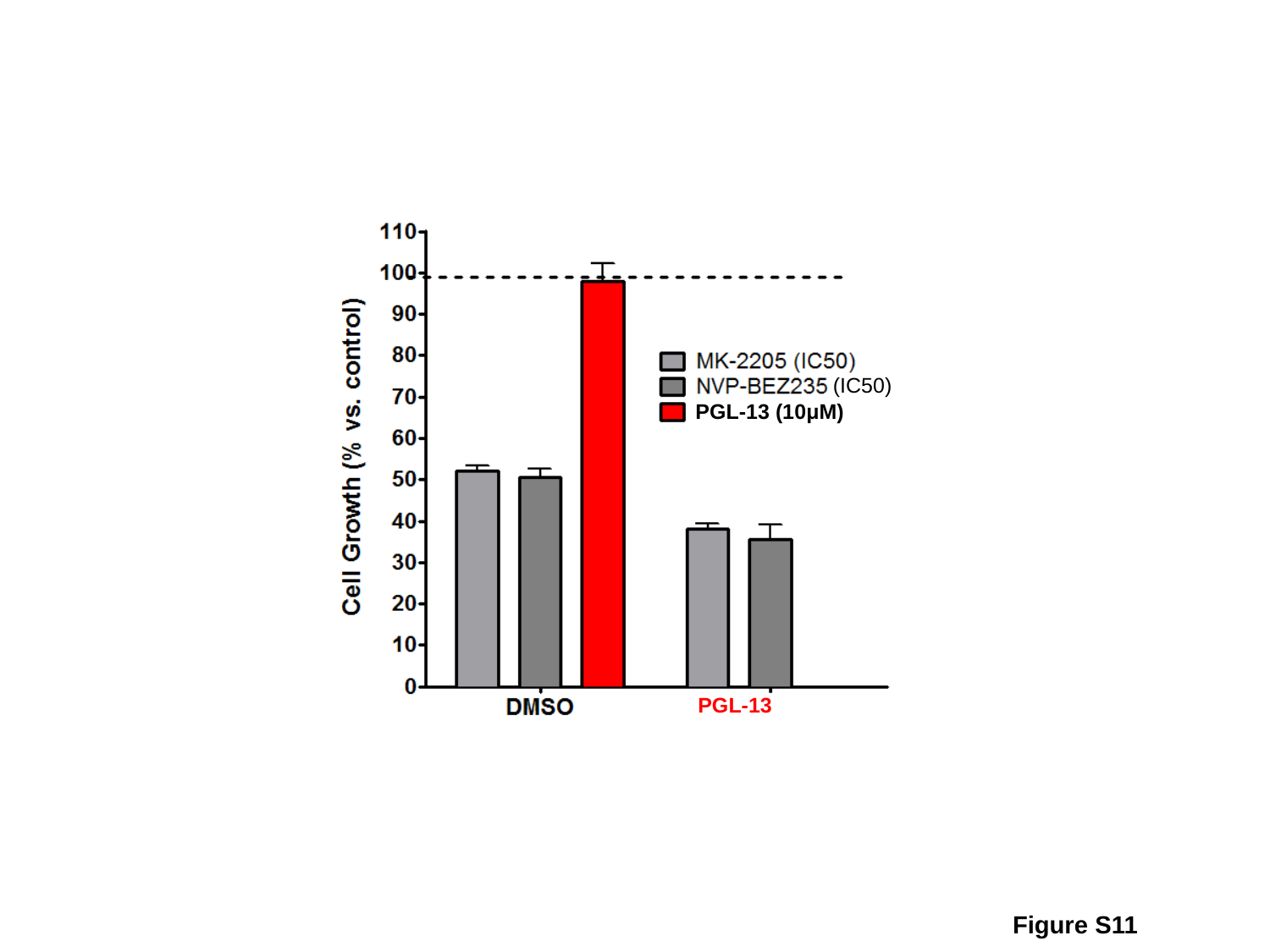

(IC50)
PGL-13 (10μM)
PGL-13
Figure S11
