# Supplementary material for: Phospho-Akt overexpression is prognostic and can be used to tailor the synergistic interaction of Akt inhibitors with gemcitabine in pancreatic cancer
Source: J Hematol Oncol. 2017 Jan 6;10:9. doi: 10.1186/s13045-016-0371-1 (PMC5219723; doi:10.1186/s13045-016-0371-1)
Supplement: Additional file 2: — Table S1. Effects of gemcitabine and perifosine and their combination on cell cycle distribution and on cell death (sub-G1). (DOCX 14 kb) [file 13045_2016_371_MOESM2_ESM.docx]

| **Table S1.** Effects of gemcitabine and perifosine and their combination on cell cycle distribution and on cell death (Sub-G1) | | | | | |
| --- | --- | --- | --- | --- | --- |
|  | **Treatment** | **G0/G1**  **Phase (%)** | **S**  **Phase (%)** | **G2/M**  **Phase (%)** | **Sub-G1** |
| **LPC0028** | Control | 54.1±1.5 | 18.7±1.7 | 27.1±2.0 | 1.9±0.1 |
|  | Gemcitabine | 44.4±3.4 | 18.8±2.5 | 36.9±3.3 | 2.6±0.2 |
|  | Perifosine | 51.2±1.8 | 26.1±1.4 | 29.7±1.7 | 9.3±1.1* |
|  | Gemcitabine + Perifosine | 36.9±0.9 | 48.9±0.6 | 30.2±2.1 | 17.6±1.2* |
| **CFPAC-1** | Control | 57.5±1.2 | 9.2±1.3 | 33.3±1 | 1.9±0.1 |
|  | Gemcitabine | 43.2±1.4 | 24.6±1.1 | 32.2±1.4 | 4.8±0.5 |
|  | Perifosine | 16.3±0.8 | 59.1±1.4 | 24.6±0.3 | 9.2±1.1* |
|  | Gemcitabine + Perifosine | 11.9±2.2 | 41.2±2.7 | 28.8±1.2 | 15.8±1.2* |
| **LPC006** | Control | 52.4±2.3 | 25.8±1.9 | 21.8±0.3 | 2.1±0.6 |
|  | Gemcitabine | 38.3±1.5 | 30.1±2.1 | 31.6±1.9 | 11.9±0.8* |
|  | Perifosine | 55.0±2.1 | 22.3±1.9 | 22.6±1 | 4.3±1.1 |
|  | Gemcitabine + Perifosine | 57.1±1.8 | 24.5±2.6 | 18.4±0.6 | 10.7±2.3* |
| **PANC-1** | Control | 55.2±0.9 | 17.7±1.3 | 27.2±2.2 | 3.2±0.4 |
|  | Gemcitabine | 46.4±2.8 | 17.8±2.8 | 35.9±5.6 | 8.6±1.2* |
|  | Perifosine | 51.0±2.4 | 23.1±1.9 | 25.9±1.1 | 10.3±1.2* |
|  | Gemcitabine + Perifosine | 32.2±0.8 | 47.0±2.1 | 30.8±2.1 | 18.9±3.2* |
| Note. Cells were exposed to IC50s values of gemcitabine, perifosine and their combination, for 72 hours. *Statistically significant compared to control (P<0.05) | | | | | |
